# Long-read sequence assembly of the firefly *Pyrocoelia pectoralis* genome

**DOI:** 10.1093/gigascience/gix112

**Published:** 2017-11-24

**Authors:** Xinhua Fu, Jingjing Li, Yu Tian, Weipeng Quan, Shu Zhang, Qian Liu, Fan Liang, Xinlei Zhu, Liangsheng Zhang, Depeng Wang, Jiang Hu

**Affiliations:** Hubei Insect Resources Utilization and Sustainable Pest Management Key Laboratory, College of Plant Science and Technology, Huazhong Agricultural University, Shizishan Street, Hongshan District, Wuhan, Hubei 430000, China; Nextomics Biosciences Institute, Biolake, No. 666 Gaoxin Road, Wuhan, Hubei 430000, China; Firefly Conservation Research Centre, Shizishan Street, Hongshan District, Wuhan, Hubei 430000, China; Institute for Genomic Medicine, Columbia University, 116th Street and Broadway, New York, NY 10032, USA; Center for Genomics and Biotechnology, State Key Laboratory of Ecological Pest Control for Fujian and Taiwan Crops, Fujian Agriculture and Forestry University, Shangxiadian Road, Cangshan District, Fuzhou 350002, China

**Keywords:** firefly, *Pyrocoelia pectoralis*, genome, long reads, assembly

## Abstract

**Background:**

Fireflies are a family of insects within the beetle order Coleoptera, or winged beetles, and they are one of the most well-known and loved insect species because of their bioluminescence. However, the firefly is in danger of extinction because of the massive destruction of its living environment. In order to improve the understanding of fireflies and protect them effectively, we sequenced the whole genome of the terrestrial firefly *Pyrocoelia pectoralis*.

**Findings:**

Here, we developed a highly reliable genome resource for the terrestrial firefly *Pyrocoelia pectoralis* (E. Oliv., 1883; Coleoptera: Lampyridae) using single molecule real time (SMRT) sequencing on the PacBio Sequel platform. In total, 57.8 Gb of long reads were generated and assembled into a 760.4-Mb genome, which is close to the estimated genome size and covered 98.7% complete and 0.7% partial insect Benchmarking Universal Single-Copy Orthologs. The k-mer analysis showed that this genome is highly heterozygous. However, our long-read assembly demonstrates continuousness with a contig N50 length of 3.04 Mb and the longest contig length of 13.69 Mb. Furthermore, 135 589 SSRs and 341 Mb of repeat sequences were detected. A total of 23 092 genes were predicted; 88.44% of genes were annotated with one or more related functions.

**Conclusions:**

We assembled a high-quality firefly genome, which will not only provide insights into the conservation and biodiversity of fireflies, but also provide a wealth of information to study the mechanisms of their sexual communication, bio-luminescence, and evolution.

## Data Description

### Background

Fireflies (Coleoptera: Lampyridae) are the best-known example of a species that displays bioluminescence. They produce a cold light in a specific stage of development. With more than 2000 species in 100 genera, worldwide, lampyrid biodiversity is impressive and includes diurnally active as well as nocturnal species [1]. Most firefly species are terrestrial, and only 9 species are aquatic [2]. The terrestrial firefly *P. pectoralis* is widely distributed in mainland China. Larval *P. pectoralis* has been reported as a major predator of land snails and has been suggested as a possible bio-control agent to control snail species [3]. Adults emerge in October and are sexually dimorphic. Flightless females glow sedentarily and release sex pheromones to attract flying and glowing males to mate [4]. However, water pollution, habitat conversion, agricultural chemical run-off, artificial light pollution, and commercial harvesting and trade pose major threats to fireflies [5]. Populations of many species of fireflies have declined rapidly in the world, especially aquatic species that are most sensitive to water quality and pollution. Conservation of fireflies as an enigmatic umbrella species can have a great impact in protecting bio-diversity and also could be a good way to conduct sustainable community development as eco-tourism. However, even with so many species of lampyridae, the genetic basis and the evolutionary characteristics of lampyridae are still unclear, and very little information about fireflies is available in public databases. In order to improve the understanding of fireflies and explore the mechanisms of complex traits of their life history, we sequenced the firefly genome.

### Sampling and sequencing

**Figure 1:**
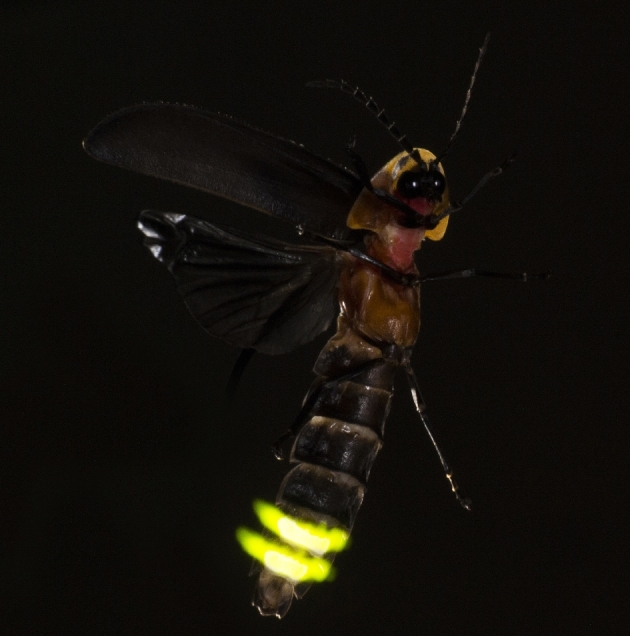
Example of *P. pectoralis* (image from Xinhua Fu).

Genomic DNA was extracted [6] from a female adult *P. pectoralis* (NCBI taxonomy ID: 417401) (Fig. 1) that was bred at the College of Plant Science and Technology, Huazhong Agricultural University (Accession number PP01), from a wild larvae collected from the field (Xianjian Village, Hongshan District, Wuhan 430070, Hubei, China). Two libraries with insert sizes of 400 bp and 20 kb were constructed using Illumina TruSeq Nano DNA Library Prep Kits and SMRTbell Template Prep Kits separately. The short insert size (400 bp) library was sequenced on an Illumina HiSeq X Ten instrument at Genetron Health (Beijing, China) using a whole-genome shotgun sequencing (WGS) strategy, and a total of 47.4 Gb of raw data was collected (Table S1). For the long insert size (20 kb) library, we sequenced it on a PacBio Sequel instrument with Sequel SMRT cells 1M v2 (Pacific Biosciences p/n101–008-000) with 1 movie of 600 minutes at the Genome Center of Nextomics (Wuhan, China) and obtained 57.8 Gb of long reads (polymerase reads) data (Table S1); The average length and the N50 of long subreads are 9.5 kb and 15.6 kb, respectively (Fig. S1).

The raw data were filtered using different strategies based on the sequencing platform to reduce low-quality bases or reads. For the Illumina data, we used the following strategies to filter raw data [7]: (i) filtered reads with adapters; (ii) trimmed reads with 2 low-quality bases at the 5’ end and 3 low-quality bases at the 3’end; (iii) filtered reads with N bases more than 10%; (iv) filtered duplicated reads due to polymerase chain reaction amplification; (v) filtered reads with low-quality bases (≤5) greater than 50%. For the PacBio data, subreads were filtered with the default parameters. Finally, we obtained 41.9 Gb of short clean reads and 57.7 Gb of long reads, respectively, which were used for further downstream analyses.

### Assembly and correction

The genome size was estimated based on the k-mer spectrum [8]: G = (K_total_– K_error_)/D, where K_total_ is the total count of k-mers, K_error_ is the total count of low-frequency (frequency ≤ 1) k-mers that are probably caused by sequencing errors, G is the genome size, and D is the k-mer depth. Using Jellyfish (v2.1.3) [9], 17-mers were counted as 37 238 236 952 from short clean reads. The total count of error kmers was 1 144 064 507, and the kmer depth was 46 (Fig. S2). Therefore, the genome size of *P. pectoralis* was estimated to be approximately 785 Mb.

Falcon (v0.4) [10] was used for genome assembly. Falcon is a hierarchical genome assembly process assembler, which is specifically designed to perform *de novo* assembly for PacBio long reads with about 15% random errors [11]. The *de novo* assembly of PacBio long reads was generated by executing the following steps: (i) raw subreads overlapping for error correction; (ii) pre-assembly and error correction; (iii) overlapping detection of the error corrected reads; (iv) overlap filtering; (v) constructing a graph from the overlaps; (vi) constructing a contig from the graph. After error correction, where a length cutoff of 9 kb was used for initial seed reads mapping, we obtained about 36 Gb of error-corrected reads (10.3 kb average length and 13.9 kb N50); Then the error-corrected reads were used to construct an assembly graph with the following parameters: length_cutoff_pr = 15 000, max_diff = 60, max_cov = 60, min_cov = 2, and the end assembly result was 1.1 Gb, and N50 was 2.3 Mb (Table 1).

**Table 1:** Comparison of genome features between *P. pectoralis* an*d D. melanogaster*

Type	Original assembly	Filtered assembly	*D. melanogaster*
Total number	3517	474	2442
Total length, bp	1119 821 639	760 416 098	142 573 024
Average length	318 403	1604 253	58 384
N50 length, bp/number	2316 748/136	3035 809/79	21 485 538/3
N90 length, bp/number	161 781/689	813 338/261	666 663/17
Longest	13 688 299	13 688 299	27 905 053
GC content, %	34.69	34.79	42.01
BUSCO (n = 1658)	C: 98.8%, F: 0.6%,	C: 98.7%, F: 0.7%	C: 99.7%, F: 0.2%

C: complete BUSCOs; F: fragmented BUSCOs.

To further improve the accuracy of the reference assembly, 2 steps of polishing strategies were performed for the initial assembly. Initial polishing was performed with Arrow [12] using PacBio long reads only. Arrow, as a successor of Quiver [12], employs an improved consensus model based on a more straightforward hidden Markov model approach. This step corrected 3 150 957 insertions, 416 262 deletions, and 515 012 substitutions. Because of the high error rate of PacBio raw reads, we also used Pilon v1.20 (Pilon, RRID:SCR_014731) [13] to further correct the PacBio-corrected assembly with the highly accurate Illumina short reads. The result showed that 158 401 insertions, 25 390 deletions, and 10 884 substitutions were corrected in this step. Finally, we used BWA v0.7.12 (BWA, RRID:SCR_010910) [14] to map short reads to the error-corrected assembly. Then SAMtools v0.1.19 (SAMtools, RRID:SCR_002105) [15] and FreeBayes v0.9.14 (FreeBayes, RRID:SCR_010761) [16] with default parameters under the diploid model were applied to call homozygous variations to calculate an estimated quality value. The rate of homozygous variation site is about 1.8*10^−6^ (QV47), suggesting that our assembly is highly accurate at the base level.

### Filter heterozygous and contaminated contigs

**Figure 2:**
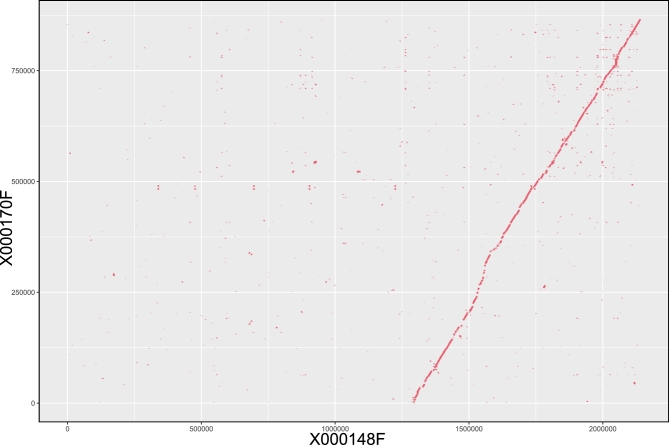
A demo of filtering heterozygous contigs. The alternative heterozygous regions between contig X000148F (x-axis) and contig X000170F (y-axis) are represented by red lines. The breakpoints of the main red line are caused by highly heterozygous loci. In total, 83.49% of short contig X000170F (865 792 bp) was covered by long contig X000148F (2 140 267 bp) with identity 0.94, so the short one was removed and the long contig was kept in the finally assembly.

Recent publications [10, 17–19] showed that a standard assembly process tends to collapse homozygous regions and report heterozygous regions in alternative contigs for a high heterozygous genome, as the heterozygous characteristics can result in a chimeric genome assembly and the assembly genome size will be larger than expected and also lead to a loss of polymorphic information in heterozygous regions. For the *P. pectoralis* genome, the assembly genome size (1.1 Gb) was 315 Mb larger than the genome size (785 Mb) estimated in 17-mer analysis (Fig. S2, Table 1), in addition, 17-mer analysis showed that this genome was a highly heterozygous genome (Fig. S2). Considering these factors, we considered that this assembly contained 2 or more copies for heterozygous regions of the firefly genome. To resolve the haplotype genome and to overcome the bias for further analysis, we employed a whole-genome alignment (WGA) strategy to recognize and selectively remove alternative heterozygous contigs. First, we used MUMmer v3.23 (–mumreference -b 500 -g 200 -l 100) [20] and Last (v864) [21] to do the whole-genome self-alignment to remove single software bias. Because the firefly genome was highly heterozygous, the alignment result was fractional even for the same loci in homologous chromosomes. Mummer prefers to find a series of consecutive matches and break at a high heterozygous region; Thus we used longest increasing subset algorithm (LIS) [22] to cluster small individual matches into larger matches. While Last tends to find all short matches and give a redundant result, we used a merge strategy [19] that filtered repeat alignments by alignment scores and then merged adjacent match blocks. We calculated the coverage of overlap length for each pair of contigs and discarded the short one if 80% of the total length was aligned to the long contig (Fig. 2). For each removed redundant contig, we also generated a dot plot to examine possible alignment errors and restored the removed contigs if the alignment quality was poor.

Mitochondrial contigs were removed by aligning to mitochondrial references of firefly; any contigs with 80% of the total length aligned to mitochondrial references with E-value less than 1e-5 were discarded as mitochondrias. Potential contaminated contigs were identified by using taxon-annotated GC coverage (TAGC) plots with BlobTools (v1.0) [23] under the “bestsumorder” rule. Contigs with coverage below 10 on the blobplot or that had the best hit to non-Arthropoda and without any transcript reads and homolog genes from Benchmarking Universal Single-Copy Orthologs v2.0 (BUSCO, RRID:SCR_015008) [24] maps were discarded from further analysis (Fig. S3, Table S4). Finally, we obtained a 760.4-Mb assembly genome, representing 96.9% of the estimated genome size, with contig N50 length of 3.04 Mb and the longest contig length 13.69 Mb (Table 1).

### Assessment of genome completeness

**Figure 3:**
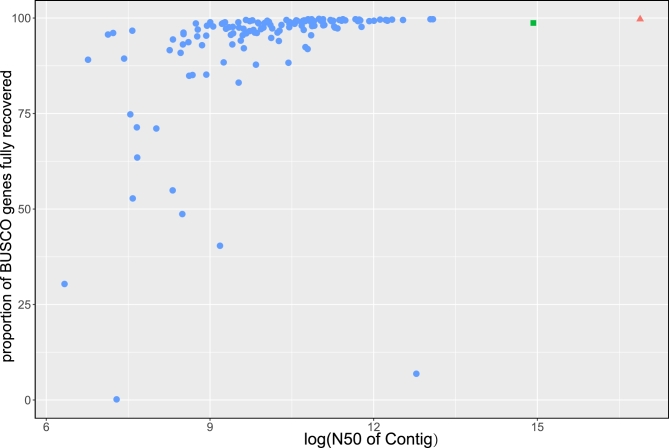
The quality of genome assembly of 137 insects. The completeness of genome assemblies (y-axis) was assessed using 1658 insecta BUSCOs. The x-axis is the contig N50 (bp) of different insect genomes with log transformation to reduce the range. The red triangle and green square represent the *D. melanogaster* genome and *P. pectoralis* genome, respectively. The blue points represent 135 other insect genomes.

The completeness of the assembly was evaluated by BUSCO (v3.0) and transcriptomic reads (downloaded from NCBI, accession SRX2036804). The result of BUSCO analysis proved that our assembly covered 98.7% complete and 0.7% partial insect BUSCOs, with only 0.6% missed (Table 1). Comparing our assembly with other published insect genomes (data from InsectBase) [25], the contig N50 length of our assembly was the longest, except for model insect *Drosophila melanogaster* [26], while the result of BUSCO analysis corresponded closely to *D. melanogaster* (Fig. 3). The contig number of our assembly was less than *D. melanogaster*, and the average length of contigs was about 27-fold longer than that of *D. melanogaster* (Table 1). When mapping the transcriptomic reads and unigenes assembled with Trinity v20140717 (Trinity, RRID:SCR_013048) [27] to our assembly genome using histat2 (v2.05) [28] and Blat [29], about 98% unigenes and 90% reads could be mapped to the assembly genome (Table 2, Table S2). For the unmapped reads and unigenes, we speculated this was caused by high heterozygosity between different individuals. In summary, all the results suggested that the quality, including base level accuracy and completeness of our assembly, was high for our reference genome for the firefly (Fig. 3, Table 1).

**Table 2:** The coverage of unigenes from *P. pectoralis*

					Coverage rate		Coverage rate	
					>90% in 1 contig		>50% in 1 contig	
			Total length,	Sequence covered by				
Data set		Number	bp	assembly (%)	Number	Percentage	Number	Percentage
Original assembly	All	37 552	30 971 346	98.28	34 963	93.10	36 636	97.56
	>500 bp	15 237	24 436 334	99.35	14 521	95.30	15 050	98.77
	>1000 bp	9041	20 067 802	99.77	8730	96.56	8980	99.32
Filtered assembly	All	37 552	30 971 346	97.88	34 472	91.79	36 389	96.90
	>500 bp	15 237	24 436 334	99.11	14 387	94.42	14 979	98.30
	>1000 bp	9041	20 067 802	99.60	8668	95.87	8950	98.99

### Repeat analysis

Simple sequence repeats (SSRs) are repeating sequences of 1–6 base pairs of DNA that exist extensively in genomes. We identified SSRs in the firefly genome with the MIcroSAtellite identification tool (MISA, RRID:SCR_010765) [30], which can identify and locate simple microsatellites such as 10 repeats for mono-, 6 repeats for di-, and 5 repeats for tri-, tetra-, penta-, hexa-, and hepta-nucleotide, as well as compound microsatellites, which are interrupted by a certain number of bases. In total, 135 589 SSRs were found in the *P. pectoralis* genome, and the most SSRs with repeat unit constitutes of 2 or more bases was (AAT)_5_, while the most abundant repeat unit with 2 or more bases was TAT (Table S3). This was different from the genome of *Tribolium castaneum* [31], one of another coleoptera genomes, (AAT)_5_, and its repeat unit, AAT, were the most SSR and repeat unit, respectively. We selected 2237 SSRs (Additional file 2), which can be used as genetic markers in population genetic studies according to the following criteria: (i) perfect repeats with the minimum number of repeat units for di-, tri-, and tetra-nucleotide were 6, 5 and 5, respectively; (ii) no SSRs located within 2 kb upstream and downstream flanking regions; (iii) filtered SSRs located in the repeat regions; (iv) 200-bp upstream and downstream flanking sequences cannot be mapped to other positions of the reference genome.

Repetitive sequences including tandem repeats and transposable elements (TEs) were searched for the *P. pectoralis* genome. First, we used tandem repeats finder (TRF, v4.07b) [32] to annotate the tandem repeats with the following parameters: 2 7 7 80 10 50 2000. About 3.73% of the *P. pectoralis* genome was identified as tandem repeats. TEs were identified using a combination of de *novo* and homology-based approaches at both the DNA and protein levels. At the DNA level, we used RepeatModeler v1.0.8 (RepeatModeler, RRID:SCR_015027) [33] to construct a *de novo* repeat library, which built a repeat consensus database with classification information, and we adopted RepeatMasker v4.0.6 (RepeatMasker, RRID:SCR_012954) [33] to search similar TEs against the known Repbase TE library (Repbase21.08) [34] and *de novo* repeat library. At the protein level, RepeatProteinMask within the RepeatMasker package (v4.0.6) was used to search against the TE protein database using a WU-BLASTX engine. Overall, the *P. pectoralis* genome comprised approximately 44.88% repetitive sequences, and 60.68% of repetitive sequences were TEs. DNA transposons accounted for 15.25% of the *P. pectoralis* genome (Table 3), representing the most abundant repeat class.

**Table 3:** Summary statistics of annotated repeats

	Number of	Length	Percentage of
Type	elements	occupied, bp	sequence
DNA	292 513	115 966 469	15.25
LINE	156 922	63 646 057	8.37
SINE	4935	634 774	0.08
LTR	35 391	26 864 897	3.53
Other	96 807	39 411 289	5.18
Unknown	384 377	99 828 399	13.13
Total	970 945	341 311 350	44.88

### Gene prediction

Gene models were constructed with MAKER v.2.31.8 (MAKER, RRID:SCR_005309) [35], which incorporates *ab initio* prediction, homology-based prediction, and RNA-seq-assisted prediction. For *ab initio* gene prediction, repeat regions of the *P. pectoralis* genome were first masked based on the result of repeat annotation, and then SNAP (V2006–07-28) [36], GeneMark (v4.32) [37], and Augustus v3.2.2 (Augustus: Gene Prediction, RRID:SCR_008417) [38], trained for model parameters from homologous genes of BUSCOs, were employed to generate gene structures. For homology-based prediction, protein sequences from 5 sequenced insects, *T. castaneum* [31]*, D. melanogaster* [26]*, Apis mellifera* [39]*, Acyrthosiphon pisum* [40]*, Pediculus humanus* [41], and *Homo sapiens* (downloaded from the Ensembl database), were initially mapped onto the *P. pectoralis* genome using tBlastn [42]. Subsequently Exonerate (v2.2.0) [43] was used to polish BLAST hits to get exact intron/exon positions. Furthermore, 8 tissues of *P. pectoralis* and published *P. pectoralis* transcriptomic data (downloaded from NCBI, accession SRX2036804) [44] assembled with Histat2 (v2.05) and Trinity (v20140717) were used to identify candidate exon regions and donor and acceptor sites. Finally, all predictions were integrated to produce a consensus gene set. The gene set was aligned to the transposon database by TransposonPSI (v08222010) [45] with default parameters. Any gene homology to transposons was removed in the final gene set. In total, 23 092 protein-coding genes were identified in *P. pectoralis* genome (Table 4). Compared with other existing published coleoptera genomes, the number of genes in *P. pectoralis* corresponds to that of *Anoplophora glabripennis* (22 035 genes) [46], while the gene number is greater than *T. castaneum* (16 526 genes) [31].

**Table 4:** Summary statistics of genes and function annotation

	Number	Percentage
Type	of genes	of genes
InterProScan	18 318	79.33
GO	12 648	54.77
KEGG	7930	34.34
Swissprot	15 813	68.48
Trembl	20 061	86.87
Annotated	20 423	88.44
Total	23 092	100.00

### Functional annotation of protein-coding genes

Gene functions were assigned according to the best match by aligning protein sequences predicted from the *P. pectoralis* genome to SwissProt and TrEMBL databases [47] using Blastp (with a threshold of E-value ≤ 1e-5), and KAAS (v2.1) [48] was used to extract the pathway in which the gene might be involved. Motifs and domains were annotated using InterProScan v5.24 (InterProScan, RRID:SCR_005829) [49] by searching against publicly available databases including ProDom (ProDom, RRID:SCR_006969), PRINTS (PRINTS, RRID:SCR_003412), Pfam (Pfam, RRID:SCR_004726), SMRT, PANTHER (PANTHER, RRID:SCR_004869), and PROSITE (PROSITE, RRID:SCR_003457). The Gene Ontology [50] IDs for each gene were assigned by the corresponding InterPro entry. In summary, 20 423 genes were annotated with at least 1 related function, which accounted for about 88.44% of the genes of *P. pectoralis* (Table 4).

## Conclusion

Here we report the first genome of Lampyridae, which is a high-quality reference genome for the firefly. This genome provides a core resource to study the mechanisms of complex traits such as the sexual communication and bio-luminescence of fireflies, and it can be used to give a better protection for the bio-diversity of fireflies. It also fills a gap for large-scale phylogenomic projects such as i5K and 1KITE to study the evolution of insects.

## Availability of supporting data

Raw sequencing reads have been deposited in the Sequence Read Archive database with Bioproject ID PRJNA394639. The genome assembly, gene models, and SSRs with flanking sequences, and other supporting data, are available via the *GigaScience* database, *Giga*DB [51]. The DNA extraction protocol is available via protocols.io [6].

## Additional files

Additional file 1: Supplementary Figures and Tables.docx.

Additional file 2: SSR.xls.

## Abbreviations

BUSCO: Benchmarking Universal Single-Copy Orthologs; SMRT: single molecule real time; SRA: Sequence Read Archive; SSR: simple sequence repeats; TAGC: taxon annotated GC coverage; TE: transposable element; TRF: tandem repeats finder; WGS: whole-genome shotgun sequencing.

## Competing interests

D.W., W.Q., J.H., J.L., S.Z., Y.T., and F.L. are employees of Nextomics Bioscences. All other authors declare that they have no competing interests.

## Author contributions

X.F., L.Z., and D.W. designed the study; X.F. and X.Z. collected samples; W.Q. extracted DNA samples and worked on sequencing; J.H, J.L., and Q.L. worked on the genome assembly; S.Z. worked on the assessment of the assembly; Y.T. and F.L. worked on annotation; J.H. and X.F. wrote the manuscript. All authors read and approved the final version of the manuscript.

## Supplementary Material

GIGA-D-17-00199_Original-Submission.pdf

GIGA-D-17-00199_Revision-1.pdf

GIGA-D-17-00199_Revision-2.pdf

Response-to-Reviewer-Comments_Original-Submission.pdf

Response-to-Reviewer-Comments_Revision-1.pdf

Reviewer-1-Report-(Original-Submission).pdf

Reviewer-2-Report-(Original-Submission).pdf

Reviewer-2-Report-(Revision-1).pdf

Reviewer-2-Report-(Revision-2).pdf

Reviewer-3-Report-(Original-Submission).pdf

Supplemental material
