# Supplementary material for: Long-read sequence assembly of the firefly Pyrocoelia pectoralis genome
Source: Gigascience. 2017 Nov 24;6(12):1–7. doi: 10.1093/gigascience/gix112 (PMC5751067; doi:10.1093/gigascience/gix112)
Supplement: GIGA-D-17-00199_Original-Submission.pdf [file gix112_giga-d-17-00199_original-submission.pdf]

GigaScience

Long-read sequence assembly of the firefly Pyrocoelia pectoralis genome

--Manuscript Draft--

Manuscript Number:	GIGA-D-17-00199
Full Title:	Long-read sequence assembly of the firefly Pyrocoelia pectoralis genome
Article Type:	Data Note
Funding Information:	National Science Foundation of China (31672349 , 31372252) — Mr Xinhua Fu
Abstract:	Fireflies are a family of insects within the beetle order Coleoptera, or winged beetles, which are one of the most well known and loved insect species because of their bioluminescence. However, the firefly is in danger of extinction because of the massive destruction of its living environment. In order to improve the understanding of fireflies and protect them effectively, we sequenced the whole genome of the terrestrial firefly Pyrocoelia pectoralis.<br>Findings<br>Here, we developed a highly reliable genome resource for the terrestrial firefly Pyrocoelia pectoralis (E. Oliv., 1883) (Coleoptera: Lampyridae) using single molecule real time (SMRT) cells on the PacBio Sequel platform. In total, 57.8Gb long reads were generated and assembled into a final size of 760.7Mb genome which is close to the estimated genome size and covered 98.7% complete and 0.7% partial insect BUSCOs. The k-mer analysis showed this genome is highly heterozygous. However, our long-read assembly demonstrates continuousness with a contig N50 length of 3.04Mb and the longest contig length of 13.69Mb. Furthermore, 135,623 SSRs and 341Mb of repeat sequences were detected. A total of 23,109 genes were predicted in which 88.45% genes were annotated with one or more related functions.<br>Conclusions<br>We assembled a high quality firefly genome, which will not only provide insights into the conservation and biodiversity of fireflies, but also provide a wealth of information to study the mechanisms of their sexual communication, bio-luminescence and evolution.
Corresponding Author:	jiang hu<br><br>CHINA
Corresponding Author Secondary Information:	
Corresponding Author's Institution:	
Corresponding Author's Secondary Institution:	
First Author:	Xinhua Fu
First Author Secondary Information:	
Order of Authors:	Xinhua Fu
	Jingjing Li
	Yu Tian
	Weipeng Quan
	Shu Zhang
	Qian Liu
	Fan Liang
	Xinlei Zhu
	Liangsheng Zhang
	Depeng Wang

Powered by Editorial Manager® and ProduXion Manager® from Aries Systems Corporation

	Jiang hu
**Order of Authors Secondary Information:**	
**Opposed Reviewers:**	Sara M Lewis, Ph.D<br>Professor, Tufts University<br>sara.lewis@tufts.edu<br>member of competition team
	Yuchi Oba, Ph.D<br>Associate Professor<br>yoba@isc.chubu.ac.jp<br>member of competition team
**Additional Information:**	
**Question**	**Response**
Are you submitting this manuscript to a special series or article collection?	No
**Experimental design and statistics**<br><br>Full details of the experimental design and statistical methods used should be given in the Methods section, as detailed in our Minimum Standards Reporting Checklist. Information essential to interpreting the data presented should be made available in the figure legends.<br><br>Have you included all the information requested in your manuscript?	Yes
**Resources**<br><br>A description of all resources used, including antibodies, cell lines, animals and software tools, with enough information to allow them to be uniquely identified, should be included in the Methods section. Authors are strongly encouraged to cite Research Resource Identifiers (RRIDs) for antibodies, model organisms and tools, where possible.<br><br>Have you included the information requested as detailed in our Minimum Standards Reporting Checklist?	Yes
**Availability of data and materials**<br><br>All datasets and code on which the conclusions of the paper rely must be either included in your submission or deposited in publicly available repositories (where available and ethically appropriate), referencing such data using a unique identifier in the references and in	Yes

the “Availability of Data and Materials” section of your manuscript.<br><br>Have you have met the above requirement as detailed in our Minimum Standards Reporting Checklist?	

Powered by Editorial Manager® and ProduXion Manager® from Aries Systems Corporation

Manuscript Click here to download Manuscript Manuscript.docx

Long-read sequence assembly of the firefly *Pyrocoelia pectoralis* genome

Xinhua Fu[1], Jingjing Li[2], Yu Tian[2], Weipeng Quan[2], Shu Zhang[2], Qian Liu[4], Fan Liang[2], Xinlei Zhu[3], Liangsheng Zhang[5], Depeng Wang[2,*] and Jiang Hu[2,*]

[*]Equally contributing corresponding authors: huj@grandomics.com; wangdp@grandomics.com;

[1]College of Plant Science and Technology, Huazhong Agricultural University, Wuhan, Hubei 430000, China

[2] Nextomics Biosciences Institute, Wuhan, Hubei 430000, China

[3]Firefly Conservation Research Centre, Wuhan, Hubei 430000, China

[4]Institute for Genomic Medicine, Columbia University, New York, NY 10032, USA

[5]Center for Genomics and Biotechnology, State Key Laboratory of Ecological Pest Control for Fujian and Taiwan Crops, Fujian Agriculture and Forestry University, Fuzhou 350002, China

Abstract

Fireflies are a family of insects within the beetle order Coleoptera, or winged beetles, which are one of the most well known and loved insect

species because of their bioluminescence. However, the firefly is in danger of extinction because of the massive destruction of its living environment. In order to improve the understanding of fireflies and protect them effectively, we sequenced the whole genome of the terrestrial firefly *Pyrocoelia pectoralis*.

Findings

Here, we developed a highly reliable genome resource for the terrestrial firefly *Pyrocoelia pectoralis* (E. Oliv., 1883) (Coleoptera: Lampyridae) using single molecule real time (SMRT) cells on the PacBio Sequel platform. In total, 57.8Gb long reads were generated and assembled into a final size of 760.7Mb genome which is close to the estimated genome size and covered 98.7% complete and 0.7% partial insect BUSCOs. The k-mer analysis showed this genome is highly heterozygous. However, our long-read assembly demonstrates continuousness with a contig N50 length of 3.04Mb and the longest contig length of 13.69Mb. Furthermore, 135,623 SSRs and 341Mb of repeat sequences were detected. A total of 23,109 genes were predicted in which 88.45% genes were annotated with one or more related functions.

Conclusions

We assembled a high quality firefly genome, which will not only provide

insights into the conservation and biodiversity of fireflies, but also provide a wealth of information to study the mechanisms of their sexual communication, bio-luminescence and evolution.

Keywords:

Firefly; *Pyrocoelia pectoralis*; Genome; Long reads; Assembly;

Data Description

Background

Commented [SE1]: Can you expand this section a little to give some insight into exactly why you sequenced this species. Where this fits in the tree of life, what related species have been species, and exactly what this data can achieve.

Commented [m2R1]: This part has been added.

Fireflies (Coleoptera: Lampyridae) are the best known examples of species displaying bioluminescence, and produce a cold light in specific stage of development. With more than 2000 species in 100 genera, worldwide, lampyrid biodiversity is impressive and includes diurnally active as well as nocturnal species [1]. Most firefly species are terrestrial and only 9 species belongs to aquatic [2]. The terrestrial firefly *P. pectoralis* is widely distributed in mainland China. Larval *P. pectoralis* has been reported to a major predator of land snails and has been suggested as a possible bio-control agent to control snail species [3]. Adults emerge in October and are sexually dimorphic. Flightless females glow sedentarily and release sex pheromones to attract flying and glowing males to mate [4]. However, water pollution, habitat conversion, agricultural chemical run-off, artificial light pollution, and harvest-trade

pose major threats to fireflies [5]. Populations of many species of fireflies have decline rapidly in the world, especially those aquatic species that are most sensitive to water quality and pollution. Conservation of fireflies as an enigmatic umbrella species can have a great impact in protecting bio-diversity and also could be a good way to conduct sustainable community development as eco-tourism. However, although with so many species of lampyridae, the genetic basis and the evolutionary characteristics of lampyridae are still unclear, and very little information about fireflies is available in public database. In order to improve the understanding of fireflies and explore the mechanisms of complex traits of their life history, we sequenced the firefly genome.

Sampling and sequencing

Genomic DNA was extracted [6] from a female adult *P. pectoralis* (NCBI taxonomy ID: 417401; Fig. 1) which was bred at the College of Plant Science and Technology, Huazhong Agricultural University (Accession number: PP01) from a wild larvae collected from the field (Xianjian Village, Hongshan District, Wuhan 430070, Hubei, China). Two libraries with insert sizes of 300bp and 20kb were constructed using Illumina TruSeq Nano DNA Library Prep Kits and SMRTbell Template Prep Kits separately. The short insert size (300bp) library was sequenced on an Illumina HiSeq X Ten instrument at Genetron Health (Beijing, China)

Commented [SE3]: Need some more methodological detail, particularly on DNA extraction protocols, and more detail on sequencing using both platforms. Ideally these could be inputted via protocols.io, or adapted from related protocols already there.

Commented [SE4]: How did you extract the DNA? Where exactly did you collect it, and is there an accession number for the sample stored anywhere?

Commented [m5R4]: This part has been added, and the protocol about DNA extraction has been uploaded to protocols.io which is available by reference 6.

using a whole genome shotgun sequencing (WGS) strategy and a total of 47.4Gb raw data was collected (Table S1). For the long insert size (20kb) library, we sequenced it on a PacBio Sequel instrument with Sequel SMRT cells 1M v2 (Pacific Biosciences p/n101-008-000) within one movie of 600 minutes at the Genome Center of Nextomics (Wuhan, China) and obtained 57.8Gb long reads (Polymerase Reads) data (Table S1), the average length and the N50 of long subreads is 9.5kb, 15.6kb respectively (Fig. S1).

The raw data was filtered using different strategies based on the sequencing platform to reduce low-quality bases or reads. For the Illumina data, we used the following strategies to filter raw data [7]: (i) filtered reads with adapters; (ii) trimmed reads with two low-quality bases at the 5'end and three low-quality bases at the 3'end; (iii) filtered reads with N bases more than 10%; (iv) filtered duplicated reads due to PCR amplification; (v) filtered reads with low-quality bases(<=5) more than 50%. For the PacBio data, subreads were filtered with the default parameters. Finally, we obtained 41.9Gb short clean reads and 57.7Gb long reads respectively, which were used for further downstream analyses.

Assembly and Correction

The genome size was estimated based on the k-mer spectrum [7]: G =

(K_{total}– K_{error})/D, where K_{total} is the total count of k-mers, K_{error} is the total count of low-frequency (frequency ≤1) k-mers that are probably caused by sequencing errors, G is the genome size and D is the k-mer depth. Using Jellyfish [8] (v2.1.3), 17-mers were counted as 3,7238,236,952 from short clean reads. The total count of error kmers was 1,144,064,507 and the kmer depth was 46 (Fig. S2). Therefore the genome size of *P. pectoralis* was estimated to be approximately 785Mb.

Falcon (v0.4) [9] was used for genome assembly. Falcon is a hierarchical genome assembly process assembler, which is specifically designed to perform de *novo* assembly for PacBio long reads with about 15% random errors [10]. The de *novo* assembly of PacBio long reads was generated by executing the following steps: (i) Raw subreads overlapping for error correction; (ii) Pre-assembly and error correction; (iii) Overlapping detection of the error corrected reads; (iv) Overlap filtering; (v) Constructing graph from overlaps; (vi) Constructing contig from graph. After error correction, where a length cutoff of 9kb was used for initial seed reads mapping, we obtained about 36Gb error-corrected reads (10.3kb average length and 13.9kb N50), then the error-corrected reads were used to construct assembly graph with the following parameters: length_cutoff_pr = 15000, max_diff=60, max_cov= 60, min_cov= 2, and the end assembly result is 1.1G and N50 is 2.3Mb (Table 1).

To further improve the accuracy of reference assembly, two steps

polishing strategies were performed for the initial assembly. Initial polishing was performed with Arrow [11] using PacBio long reads only. Arrow, as a successor of Quiver [12], employs an improved consensus model based on a more straightforward hidden Markov model approach. This step corrected 3,150,957 insertions, 416,262 deletions and 515,012 substitutions. Because of the high error rate of PacBio raw reads, we also used Pilon (v1.20) [12] to further correct the PacBio-corrected assembly with the highly accurate Illumina short reads. The result showed 158,401 insertions, 25,390 deletions and 10,884 substitutions were corrected in this step. Finally, we used SAMtools (v0.1.19) [13] and FreeBayes (v0.9.14) [14] to call homozygous variations to calculate an estimated quality value for the error-corrected assembly. The rate of homozygous variation site is about $1.8*10^{-6}$ (QV47), suggesting that our assembly is highly accurate at base level.

Filter heterozygous and contaminated contigs

Recent publications [9,15–18] showed that a standard assembly process tends to collapse homozygous regions and report heterozygous regions in alternative contigs for a high heterozygous genome, as the heterozygous characteristics can result in a chimeric genome assembly and the assembly genome size will be larger than expected and also lead to a loss of polymorphic information in heterozygous regions. For *P. pectoralis*

genome, the assembly genome size (1.1G) was 315M larger than the genome size (785M) estimated in 17-mer analysis (Fig. S2, Table 1), in addition, 17-mer analysis showed that this genome was a highly heterozygous genome (Fig. S2). Considering these factors, we considered that this assembly contained two or more copies for heterozygous regions of firefly genome. To resolve the haplotype genome and to overcome the bias for further analysis, we employed a whole genome alignment (WGA) strategy to recognize and selectively remove alternative heterozygous contigs. First, we used MUMmer v3.23 [19] (--mumreference -b 500 -g 200 -l 100) and Last (v864) [20] to do the whole genome self-alignment to remove single software bias. Because firefly genome was highly heterozygous, the alignment result was fractional even for the same loci in homologous chromosomes. Mummer prefers to find a series of consecutive matches and break at high heterozygous region, thus we used longest increasing subset algorithm (LIS) [21] to cluster small individual matches into larger matches. While Last tends to find all short matches and give a redundant result, we used a merge strategy [18] that filtered repeat alignments by alignment scores and then merged adjacent match blocks. We calculated the coverage of overlap length for each pair of contigs and discard the short one if 80% of the total length were aligned to the long contig (Fig. 2). For each removed redundant contig, we also generated a dot plot to examine possible alignment errors and restored the

removed contigs if the alignment quality was poor.

Bacterial and mitochondrial contigs were also removed by aligning to nucleotide database downloaded from National Center for Biotechnology Information (NCBI) and mitochondrial references of firefly separately. Any contig with 80% of the total length aligned to mitochondrial references or bacteria sequences with E-value less than 1e-5 and without any transcript reads mapped were discarded as mitochondrias or bacterias.

Five removed contigs (8.5M total size) with homolog genes from BUSCO (v2.0) [22] were added to the final assembly and finally, we obtained a 760.7 Mb of assembly genome, representing 96.9% of the estimated genome size, with contig N50 length of 3.04M and the longest contig length 13.69Mb (Table 1).

Assessment of genome completeness

The completeness of the assembly was evaluated by BUSCO (v3.0) and transcriptomic reads (downloaded from NCBI, accession SRX2036804). The result of BUSCO analysis proved that our assembly covered 98.7% complete and 0.7% partial insect BUSCOs, only 0.6% missed (Table 1). Comparing our assembly with other published insect genomes (data from InsectBase [24]), the contig N50 length of our assembly is the longest, except for model insect *Drosophila melanogaster* [4], while the result of

Commented [SE6]: Should do it with BUSCO v3

Commented [m7R6]: We have reanalysed with BUSCO v3.

BUSCO analysis corresponds closely to *D.melanogaster* (Fig. 3), the contig number of our assembly is less than *D.melanogaster* and the average length of contigs is about 26 fold than *D.melanogaster* (Table 1). When mapping the transcriptomic reads and unigenes assembled with Trinity (v20140717) [25] to our assembly genome using histat2 (v2.05) [26] and Blat [27], about 98% unigenes and 90% reads could be mapped to the assembly genome (Table 2, Table S2). For the unmapped reads and unigenes, we speculated this was caused by high heterozygosity between different individuals. In summary, all the results suggested that the quality including base level accuracy and completeness of our assembly is very close to a gold-standard reference genome for the firefly (Fig. 3, Table 1).

Repeat analysis

Simple Sequence Repeats (SSR) are repeating sequences of 1-6 base pairs of DNA and exist extensively in genomes. We identified SSRs for firefly genome with MIcroSAtellite identification tool (MISA) [28], which can identify and locate simple microsatellites such as ten repeats for mono-, six repeats for di-, and five repeats for tri-, tetra-, penta-, hexa- and hepta-nucleotide, as well as compound microsatellites which are interrupted by a certain number of bases. In total, 135,623 SSRs were found in *P. pectoralis* genome and the most SSRs with repeat unit constitutes of two or more bases is $(AAT)_5$, while the most abundant

repeat unit with two or more bases was TAT (Table S3), this was different from the genome of *Tribolium castaneum* [29], one of another coleoptera genomes, $(AAT)_5$ and its repeat unit, AAT, was the most SSR and repeat unit, respectively.

Repetitive sequences including tandem repeats and transposable elements (TEs) were searched for the *P. pectoralis* genome. First, we used tandem repeats finder (TRF, v4.07b) [30] to annotate the tandem repeats with parameters: 2 7 7 80 10 50 2000 and about 3.72% of the *P. pectoralis* genome was identified as tandem repeats. TEs were identified using a combination of de *novo* and homology-based approaches at both the DNA and protein levels. At the DNA level, we used RepeatModeler (v1.0.8) [31] to construct a de *novo* repeat library, which built a repeat consensus database with classification information, and we adopted RepeatMasker (v4.0.6) [31] to search similar TEs against the known Repbase TE library (Repbase21.08) [32] and de *novo* repeat library. At the protein level, RepeatProteinMask within the RepeatMasker package (v4.0.6) were used to search against the TE protein database using a WU-BLASTX engine. Overall, the *P. pectoralis* genome comprised approximately 44.87% repetitive sequences, and 60.48% of repetitive sequences were TEs, DNA transposons accounted for 15.24% of the *P. pectoralis* genome (Table 3), representing the most abundant repeat class.

Gene prediction

Gene models were constructed with MAKER (v.2.31.8) [33], which incoporating *ab initio* prediction, homology-based prediction and RNA-seq assisted prediction. For *ab initio* gene prediction, repeat regions of *P. pectoralis* genome were first masked based on the result of repeat annotation, and then SNAP (V2006-07-28) [34], GeneMark (v4.32) [35] and Augustus (v3.2.2) [36] trained for model parameters from homolog genes of BUSCOs were employed to generate gene structures. For homology-based prediction, protein sequences from 5 sequenced insects *T.castaneum* [29], *D.melanogaster* [4], *Apis mellifera* [37], *Acyrthosiphon pisum* [38], *Pediculus humanus* [39] and *Homo sapiens*(downloaded from the Ensembl database) were initially mapped onto the *P. pectoralis* genome using tBlastn [40] and subsequently Exonerate (v2.2.0) [41] was used to polish BLAST hits to get an exact intron/exon positions. Furthermore, 8 tissues of *P. pectoralis* and a published *P. pectoralis* transcriptomic data (downloaded from NCBI, accession SRX2036804) assembled with Histat2 (v2.05) and Trinity (v20140717) were used to identify candidate exon regions, the donor, and acceptor sites. Finally, all predictions were integrated to produce a consensus gene set. Besides, the gene set was aligned to transposon database by TransposonPSI (v08222010) [42] with default parameters. Any gene homology to transposons was removed in the final gene set. In total, 23,109

Commented [SE8]: Is there a reason this is the only coleopteran you used and not the *Anoplophora glabripennis* data? For further validation are you able to display a brief phylogeny of some of these published insect and coleopteran species

Commented [m9R8]: Because *Tribolium castaneum* is a model coleopteran insect, the contig N50 is 43873bp and this genome has updated many time.
while the contig n50 of *Anoplophora glabripennis* genome is only 16.5 kb, which will lead to many gene fragments during the gene prediction step. Besides, many genomes of coleopteran constructed gene set using the gene set of *Tribolium castaneum*. if using all of these data, the annotation pipeline will enlarge the rate of error genes from *Tribolium castaneum* gene set because most gene sets from different species support the error genes. Besides, we also used 8 tissues of *P. pectoralis* and a published *P. pectoralis* transcriptomic data to identify candidate exon regions, which has similar effect with homolog-based gene prediction and these data are enough to give a high quality evidence for gene recognition.

Commented [SE10]: We don’t like “data not show”. Is this going to be released to NCBI or GigaDB?

Commented [m11R10]: Yes, we have uploaded these data to NCBI.

protein-coding genes were identified in *P. pectoralis* genome (Table 4). Compared with other existing published coleoptera genomes, the number of genes in *P. pectoralis* corresponds to that of *Anoplophora glabripennis* [43] (22,035 genes), while the gene number is greater than *T. castaneum* [29] (16,526 genes).

Functional annotation of protein-coding genes

Gene functions were assigned according to the best match by aligning protein sequences predicted from the *P. pectoralis* genome to SwissProt and TrEMBL databases [44] using Blastp (with a threshold of E-value ≤ 1e-5), and KAAS [45] (v2.1) was used to extract the pathway in which the gene might be involved. Motifs and domains were annotated using InterProScan [46] (v5.24) by searching against publicly available databases including ProDom, PRINTS, Pfam, SMRT, PANTHER, PROSITE. The Gene Ontology [47] IDs for each gene were assigned by the corresponding InterPro entry. In summary, 20,440 genes were annotated with at least one related function which accounted for about 88.45% of genes of *P. pectoralis* (Table 4).

Conclusion

Here we report the first genome of lampyridae, which is very close to a gold-standard reference genome for the firefly. This high quality genome resource provide a core resource to study the mechanisms of complex

Commented [SE12]: Can you end with a final paragraph talking about potential uses of this data. This section can also promote discussion on possible ways the data presented might be used in or have a relationship with other areas of research that may not be directly apparent in the work. E.g. please mention its potential use filling in the gaps for large-scale phylogenomic projects such as i5K and 1KITE. And is this work useful for studying the biology and evolution of luciferase?

Commented [m13R12]: This part has been added.

traits such as sexual communication, bio-luminescence of fireflies, which can be used to give a better protection for the bio-diversity of fireflies in further. It also fill the gaps for large-scale phylogenomic projects such as i5K and 1KITE to study the evolution of insects.

Availability of supporting data

Raw sequencing reads have been deposited in the SRA (Sequence Read Archive) database with Bioproject ID PRJNA394639. The assembly genome and gene models are available via the GigaScience database. DNA extraction protocol is available in protocols.io [6].

Commented [SE14]: The bioproject/raw data in NCBI needs to be live or have reviewer access. We also need to get the assemblies, gene models and annotations into our FTP servers. Please talk to our curators at database@gigasciencejournal.com to do this

Commented [m15R14]: All reads have uploaded to NCBI and can visit by reviewer / collaborator link: ftp://ftp-trace.ncbi.nlm.nih.gov/sra/review/SRP114311_20170816_055926_ffa243f771800363714f6055d9236fd6. All other datas have uploaded to Giga DB.

Additional files

Additional File 1: Supplementary Figures and Tables.docx

Abbreviations

SMRT: Single molecule real time; WGS: whole genome shotgun sequencing; SRA: Sequence read archive; TRF: Tandem repeats finder; TE: Transposable element; BUSCO: Benchmarking universal single-copy orthologs; SSR: Simple Sequence Repeats.

Competing interests

Commented [SE16]: You might want to state some of ther authors are employees of Genetron Health

Commented [m17R16]: This part has been added.

D.W., W.Q., J.H., J.L., S.Z., Y.T. and F.L. are employees of Nextomics Bioscences. All other authors declare that they have no competing interests.

Author contributions

X.F., L.Z. and D.W. designed the study; X.F. and X.Z. collected samples;

W.Q. extracted DNA samples and worked on Sequencing; J.H, J.L. and Q.L. worked on the genome assembly; S.Z. worked on the assessment of assembly; Y.T. and F.L. worked on annotation; J.H. and X.F. wrote the manuscript. All authors read and approved the final version of the manuscript.

Acknowledgements

We thank members of Huazhong Agricultural University to prepare samples. We also thank the staff in Nextomcis Biosciences who contributed to the sequencing of the firefly genome. We thank H.C. and K.W. revised the manuscript and contributed to discussion. Financial assistance was provided by the National Science Foundation of China (# 31672349 and # 31372252).

References

1. Lewis SM, Cratsley CK. Flash signal evolution, mate choice, and predation in fireflies. Annu Rev Entomol. 2008;53:293–321.

2. Fu XH, Ballantyne LA, Lambkin CL. Aquatica gen. nov. from mainland China with a description of Aquatica wuhana sp. nov.(Coleoptera: Lampyridae: Luciolinae). Zootaxa. 2010;2530:1–18.

3. Fu X, Meyer-Rochow VB. Larvae of the firefly Pyrocoelia pectoralis (Coleoptera: Lampyridae) as possible biological agents to control the land snail Bradybaena ravida. Biol. Control. 2013;65:176–83.

4. Wang Y, Fu X, Lei C, Jeng M-L, Nobuyoshi O. Biological Characteristics of the Terrestrial Firefly Pyrocoelia pectoralis (Cleoptera: Lampyridae). Coleopt. Bull. 2007;61:85–93.

5. Firebaugh A, Haynes KJ. Experimental tests of light-pollution impacts on nocturnal insect courtship and dispersal. Oecologia. 2016;182:1203–11.

6. Hu J. DNA Extraction Procedure Using SDS. 2017, protocols.io. dx.doi.org/10.17504/protocols.io.jfpcjmn.

7. Luo R, Liu B, Xie Y, Li Z, Huang W, Yuan J, et al. SOAPdenovo2: an empirically improved memory-efficient short-read de novo assembler. Gigascience. 2012;1:18.

8. Lamichhaney S, Fan G, Widemo F, Gunnarsson U, Thalmann DS, Hoeppner MP, et al. Structural genomic changes underlie alternative reproductive strategies in the ruff (Philomachus pugnax). Nat. Genet. 2016;48:84.

9. Marçais G, Kingsford C. A fast, lock-free approach for efficient parallel counting of occurrences of k-mers. Bioinformatics. 2011;27:764–70.

10. Chin C-S, Peluso P, Sedlazeck FJ, Nattestad M, Concepcion GT, Clum A, et al. Phased diploid genome assembly with single molecule real-time sequencing. Nat. Methods. 2016;13:1050.

11. Eid J, Fehr A, Gray J, Luong K, Lyle J, Otto G, et al. Real-time DNA

sequencing from single polymerase molecules. Science. 2009;323:133–8.

12. Chin C-S, Alexander DH, Marks P, Klammer AA, Drake J, Heiner C, et al. Nonhybrid, finished microbial genome assemblies from long-read SMRT sequencing data. Nat. Methods. 2013;10:563–9.

13. Walker BJ, Abeel T, Shea T, Priest M, Abouelliel A, Sakthikumar S, et al. Pilon: an integrated tool for comprehensive microbial variant detection and genome assembly improvement. PloS One. 2014;9:e112963.

14. Li H, Handsaker B, Wysoker A, Fennell T, Ruan J, Homer N, et al. The sequence alignment/map format and SAMtools. Bioinformatics. 2009;25:2078–9.

15. Garrison E, Marth G. Haplotype-based variant detection from short-read sequencing. ArXiv Prepr. ArXiv12073907. 2012;

16. Pryszcz LP, Németh T, Gácser A, Gabaldón T. Genome comparison of Candida orthopsilosis clinical strains reveals the existence of hybrids between two distinct subspecies. Genome Biol. Evol. 2014;6:1069–78.

17. Small KS, Brudno M, Hill MM, Sidow A. A haplome alignment and reference sequence of the highly polymorphic Ciona savignyi genome. Genome Biol. 2007;8:R41.

18. Gouin A, Bretaudeau A, Lemaitre C, Legeai F. Identification and correction of genome mis-assemblies due to heterozygosity. Eur. Conf. Comput. Biol. ECCB. 2014.

19. Pryszcz LP, Gabaldón T. Redundans: an assembly pipeline for highly

heterozygous genomes. Nucleic Acids Res. 2016;44:e113–e113.

20. Kurtz S, Phillippy A, Delcher AL, Smoot M, Shumway M, Antonescu C, et al. Versatile and open software for comparing large genomes. Genome Biol. 2004;5:R12.

21. Kiełbasa SM, Wan R, Sato K, Horton P, Frith MC. Adaptive seeds tame genomic sequence comparison. Genome Res. 2011;21:487–93.

22. Schensted C. Longest increasing and decreasing subsequences. Class. Pap. Comb. Springer; 2009. p. 299–311.

23. Simão FA, Waterhouse RM, Ioannidis P, Kriventseva EV, Zdobnov EM. BUSCO: assessing genome assembly and annotation completeness with single-copy orthologs. Bioinformatics. 2015;31:3210–2.

24. Yin C, Shen G, Guo D, Wang S, Ma X, Xiao H, et al. InsectBase: a resource for insect genomes and transcriptomes. Nucleic Acids Res. 2016;44:D801–7.

25. Grabherr MG, Haas BJ, Yassour M, Levin JZ, Thompson DA, Amit I, et al. Trinity: reconstructing a full-length transcriptome without a genome from RNA-Seq data. Nat. Biotechnol. 2011;29:644.

26. Kim D, Langmead B, Salzberg SL. HISAT: a fast spliced aligner with low memory requirements. Nat. Methods. 2015;12:357–60.

27. Kent WJ. BLAT—the BLAST-like alignment tool. Genome Res. 2002;12:656–64.

28. Thiel T, Michalek W, Varshney R, Graner A. Exploiting EST

databases for the development and characterization of gene-derived SSR-markers in barley (Hordeum vulgare L.). Theor. Appl. Genet. 2003;106:411–22.

29. Richards S, Gibbs RA, Weinstock GM, Brown SJ, Denell R, Beeman RW, et al. The genome of the model beetle and pest Tribolium castaneum. 2008;

30. Benson G. Tandem repeats finder: a program to analyze DNA sequences. Nucleic Acids Res. 1999;27:573.

31. Tarailo - Graovac M, Chen N. Using RepeatMasker to identify repetitive elements in genomic sequences. Curr. Protoc. Bioinforma. 2009;4.10. 1-4.10. 14.

32. Kapitonov VV, Jurka J. A universal classification of eukaryotic transposable elements implemented in Repbase. Nat. Rev. Genet. 2008;9:411–2.

33. Holt C, Yandell M. MAKER2: an annotation pipeline and genome-database management tool for second-generation genome projects. BMC Bioinformatics. 2011;12:491.

34. Korf I. Gene finding in novel genomes. BMC Bioinformatics. 2004;5:59.

35. Ter-Hovhannisyan V, Lomsadze A, Chernoff YO, Borodovsky M. Gene prediction in novel fungal genomes using an ab initio algorithm with unsupervised training. Genome Res. 2008;18:1979–90.

36. Stanke M, Keller O, Gunduz I, Hayes A, Waack S, Morgenstern B. AUGUSTUS: ab initio prediction of alternative transcripts. Nucleic Acids Res. 2006;34:W435–9.

37. Consortium HGS. Insights into social insects from the genome of the honeybee Apis mellifera. Nature. 2006;443:931.

38. Consortium IAG. Genome sequence of the pea aphid Acyrthosiphon pisum. PLoS Biol. 2010;8:e1000313.

39. Kirkness EF, Haas BJ, Sun W, Braig HR, Perotti MA, Clark JM, et al. Genome sequences of the human body louse and its primary endosymbiont provide insights into the permanent parasitic lifestyle. Proc. Natl. Acad. Sci. 2010;107:12168–73.

40. Mount DW. Using the basic local alignment search tool (BLAST). Cold Spring Harb. Protoc. 2007;2007:pdb. top17.

41. Slater GSC, Birney E. Automated generation of heuristics for biological sequence comparison. BMC Bioinformatics. 2005;6:31.

42. TransposonPSI: An Application of PSI-Blast to Mine (Retro-) Transposon ORF Homologies. http://transposonpsi.sourceforge.net/. Accessed 18 Sep 2016.

43. McKenna DD, Scully ED, Pauchet Y, Hoover K, Kirsch R, Geib SM, et al. Genome of the Asian longhorned beetle (Anoplophora glabripennis), a globally significant invasive species, reveals key functional and evolutionary innovations at the beetle–plant interface. Genome Biol.

2016;17:227.

44. Consortium U. UniProt: a hub for protein information. Nucleic Acids Res. 2014;gku989.

45. Moriya Y, Itoh M, Okuda S, Yoshizawa AC, Kanehisa M. KAAS: an automatic genome annotation and pathway reconstruction server. Nucleic Acids Res. 2007;35:W182–5.

46. Jones P, Binns D, Chang H-Y, Fraser M, Li W, McAnulla C, et al. InterProScan 5: genome-scale protein function classification. Bioinformatics. 2014;30:1236–40.

47. Ashburner M, Ball CA, Blake JA, Botstein D, Butler H, Cherry JM, et al. Gene Ontology: tool for the unification of biology. Nat. Genet. 2000;25:25.

Figure

Figure 1: Example of *P. pectoralis* (image from Xinhua Fu).

Figure 2: A demo of filtering heterozygous contigs. The alternative heterozygous regions between contig X000148F (x axis) and contig X000170F (y axis) are represented by red lines. The breakpoints of main red line are caused by highly heterozygous loci. Totally, 83.49% of short contig X000170F (865,792bp) was covered by long contig X000148F (2,140,267bp) with identity 0.94, so the short one was removed and the

long contig was kept in the finally assembly.

Figure 3: The quality of genome assembly of 137 insects. The completeness of genome assemblies (y axis) was assessed using 1658 insecta benchmarking universal single-copy orthologs (BUSCOs). The x axis is the contig N50 (bp) of different insect genomes with log transformation to reduce the range. The red triangle and green square represent the *D. melanogaster* genome and *P. pectoralis* genome, respectively. The blue points represent other 135 insect genomes.

Table

Table 1: Comparison of genome features between *P. pectoralis* and *D. melanogaster*.

Type	Original Assembly	Filtered Assembly	*D. melanogaster*
Total Number	3,517	501	2,442
Total Length (bp)	1,119,821,639	760,732,635	142,573,024
Average Length	318,403	1,518,428	58,384
N50 Nength (bp)/Number	2,316,748/136	3,035,809/79	21,485,538/3
N90 Nength (bp)/Number	161,781/689	810,702/262	666,663/17
Longest	13,688,299	13,688,299	27,905,053

GC Content(%)	34.69	34.79	42.01
BUSCO(n=1658)	C*:98.7%,F*:0.6%,	C:98.7%,F:0.7%	C:99.7%,F:0.2%

Note: C: Complete BUSCOs; F: Fragmented BUSCOs.

Table 2: The coverage of unigenes from *P. pectoralis*.

Dataset		Number	Total Length(bp)	Sequence Covered by Assembly (100%)	Coverage rate > 90% in 1 Contig		Coverage rate > 50% in 1 Contig	
					Number	Percent	Number	Percent
Original Assembly	All	37,552	30,971,346	98.28%	34,963	93.10%	36,636	97.56%
	>500bp	15,237	24,436,334	99.35%	14,521	95.30%	15,050	98.77%
	>1000bp	9,041	20,067,802	99.77%	8,730	96.56%	8,980	99.32%
Filtered Assembly	All	37,552	30,971,346	97.88%	34,472	91.79%	36,389	96.90%
	>500bp	15,237	24,436,334	99.11%	14,387	94.42%	14,979	98.30%
	>1000bp	9,041	20,067,802	99.60%	8,668	95.87%	8,950	98.99%

Table 3: Summary statistics of annotated repeats

Type	Number of elements	Length occupied(bp)	Percentage of sequence
DNA	292,515	115,966,612	15.24%
LINE	156,924	63,646,285	8.37%

SINE	4,935	634,774	0.08%
LTR	35,394	26,865,130	3.53%
Other*	96,836	39,413,327	5.18%
Unknown*	384,377	99,828,399	13.12%
Total	970,981	341,313,925	44.87%

Note:

Other: repeats are not below mentioned types.

Unknown: repeats cannot be classified basing known databases.

Most repeats fragmented by insertions or deletions have been counted as one element.

Table 4: Summary statistics of genes and function annotation

Type	Number of genes	Percent of genes
InterProScan	18,335	79.34%
GO	12,662	54.79%
KEGG	7,936	34.34%
Swissprot	15,830	68.50%
Trembl	20,078	86.88%
Annotated	20,440	88.45%
Total	23,109	100.00%

Figure 1

Click here to download Figure Figure 1.jpg

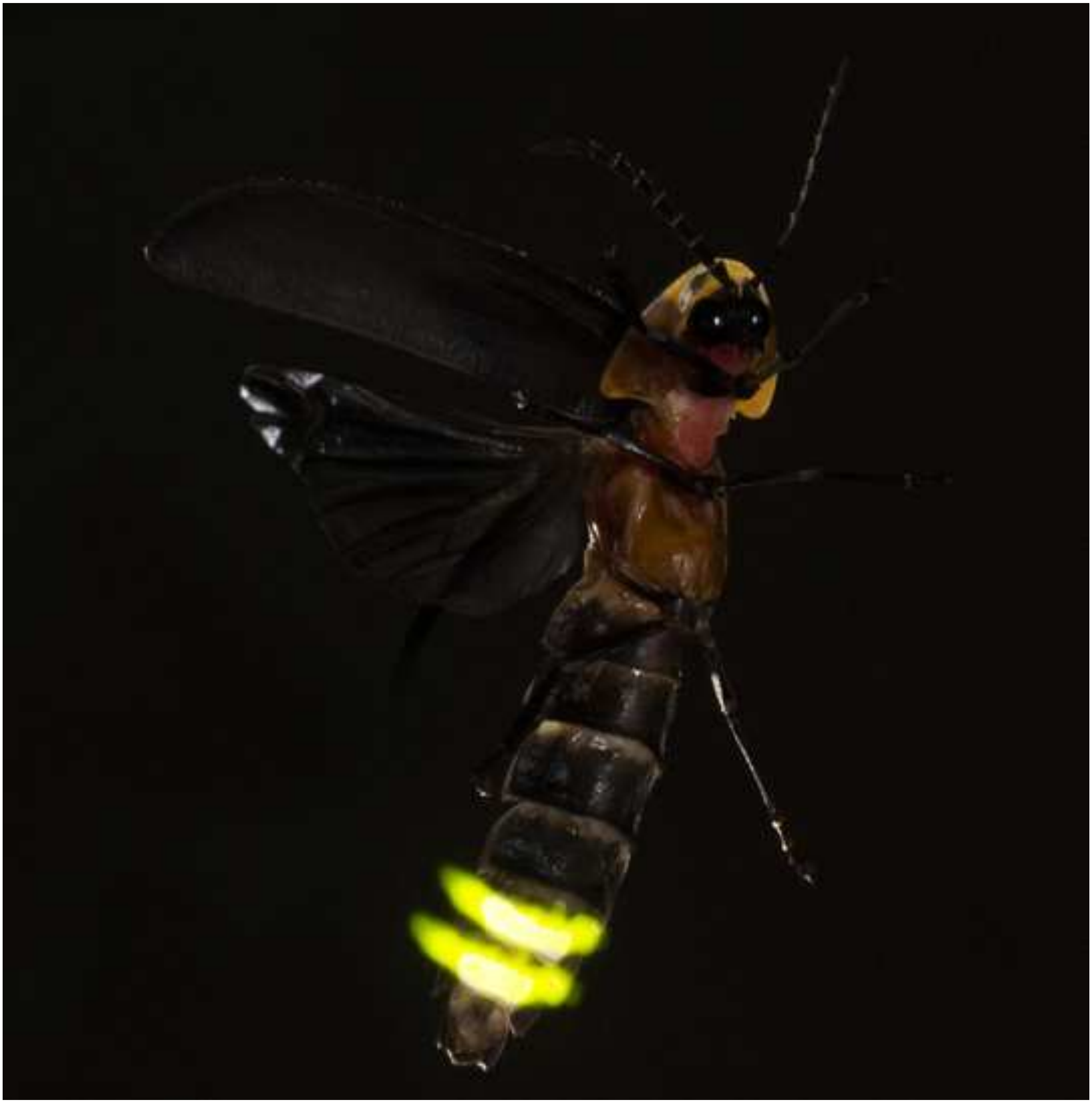

Figure 2

Click here to download Figure Figure 2.pdf

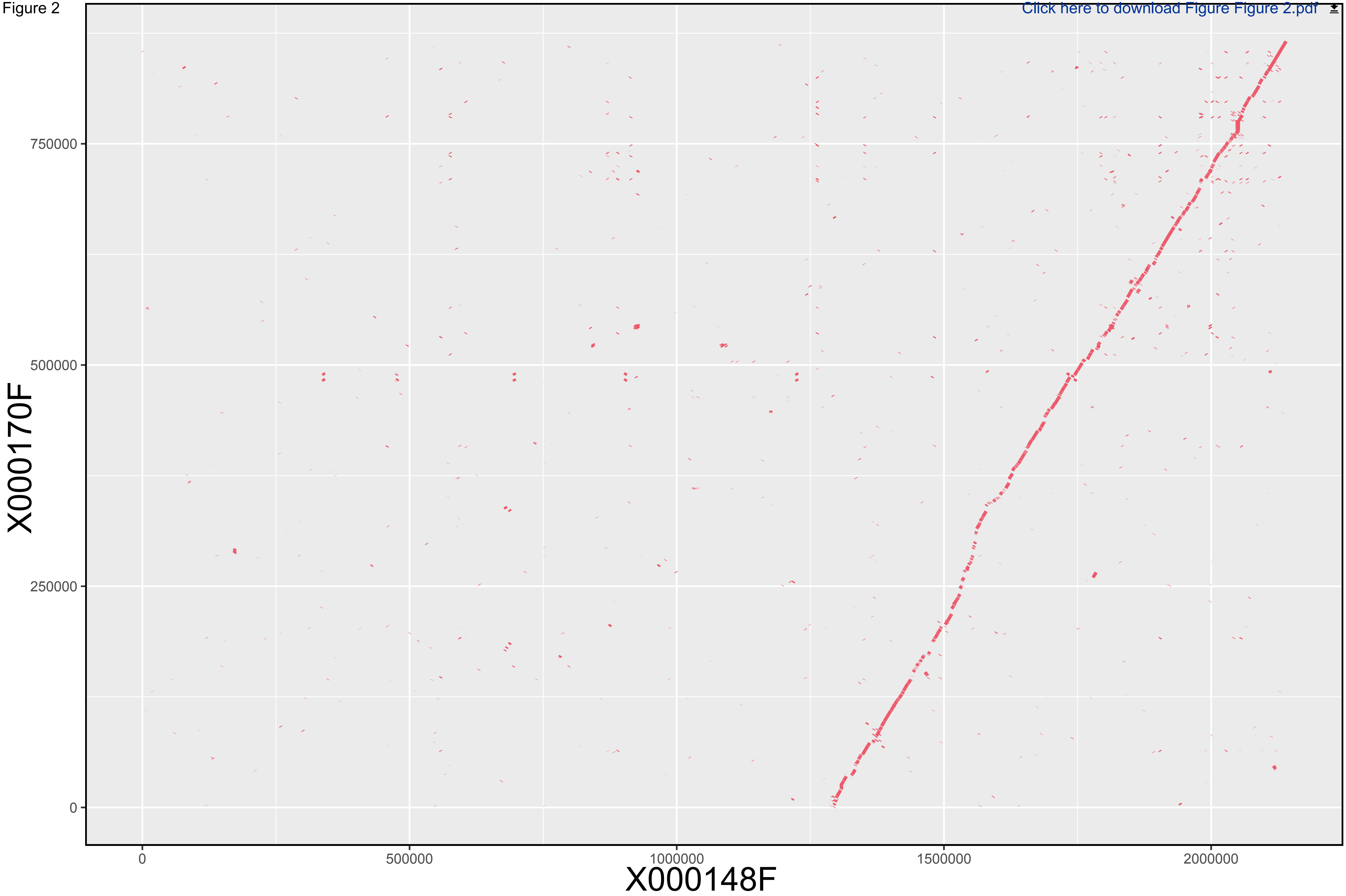

Figure 3

Click here to download Figure Figure 3.pdf

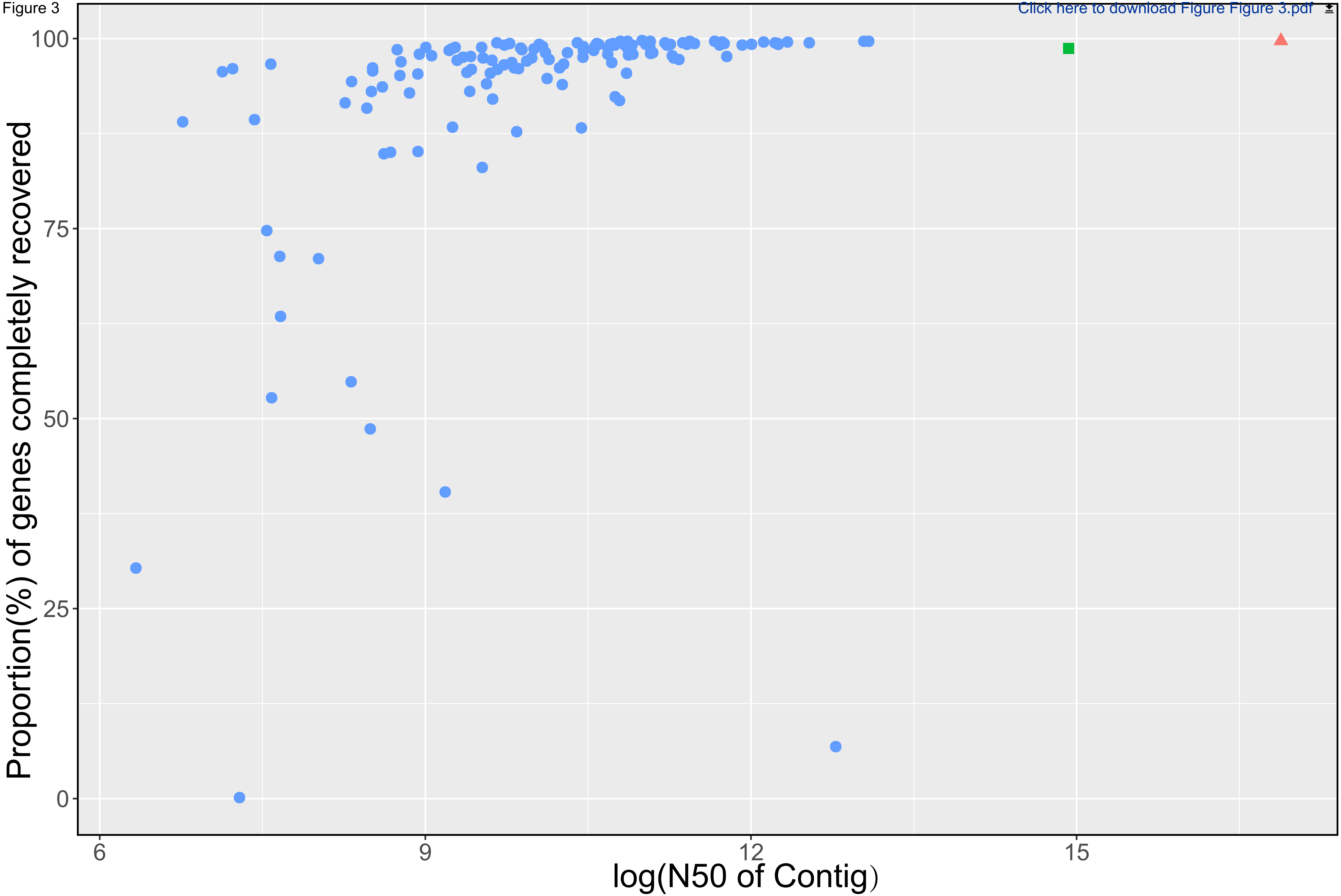

Figure S1

Click here to download Figure Figure S1.pdf

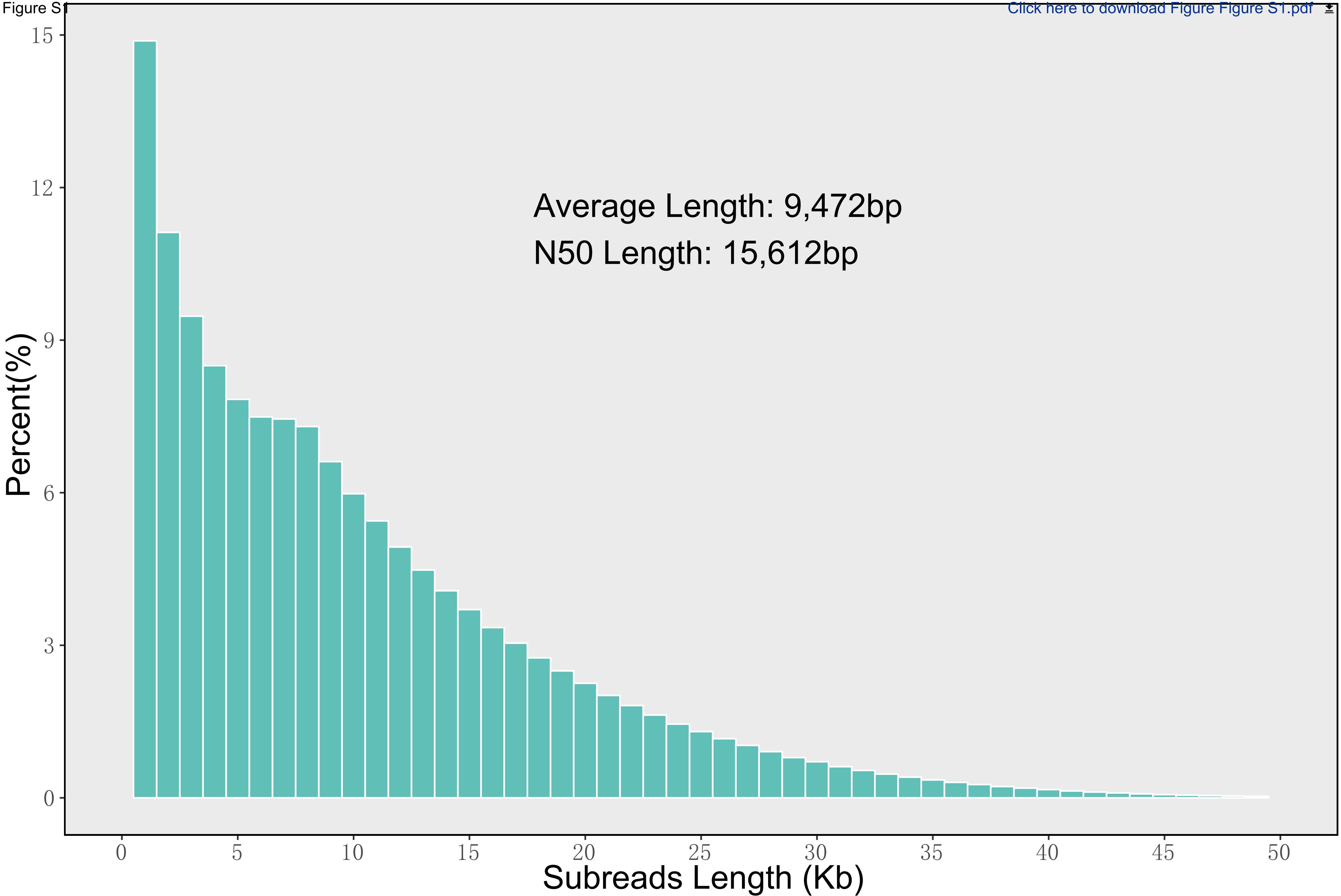

Figure S2

Click here to download Figure Figure S2.pdf

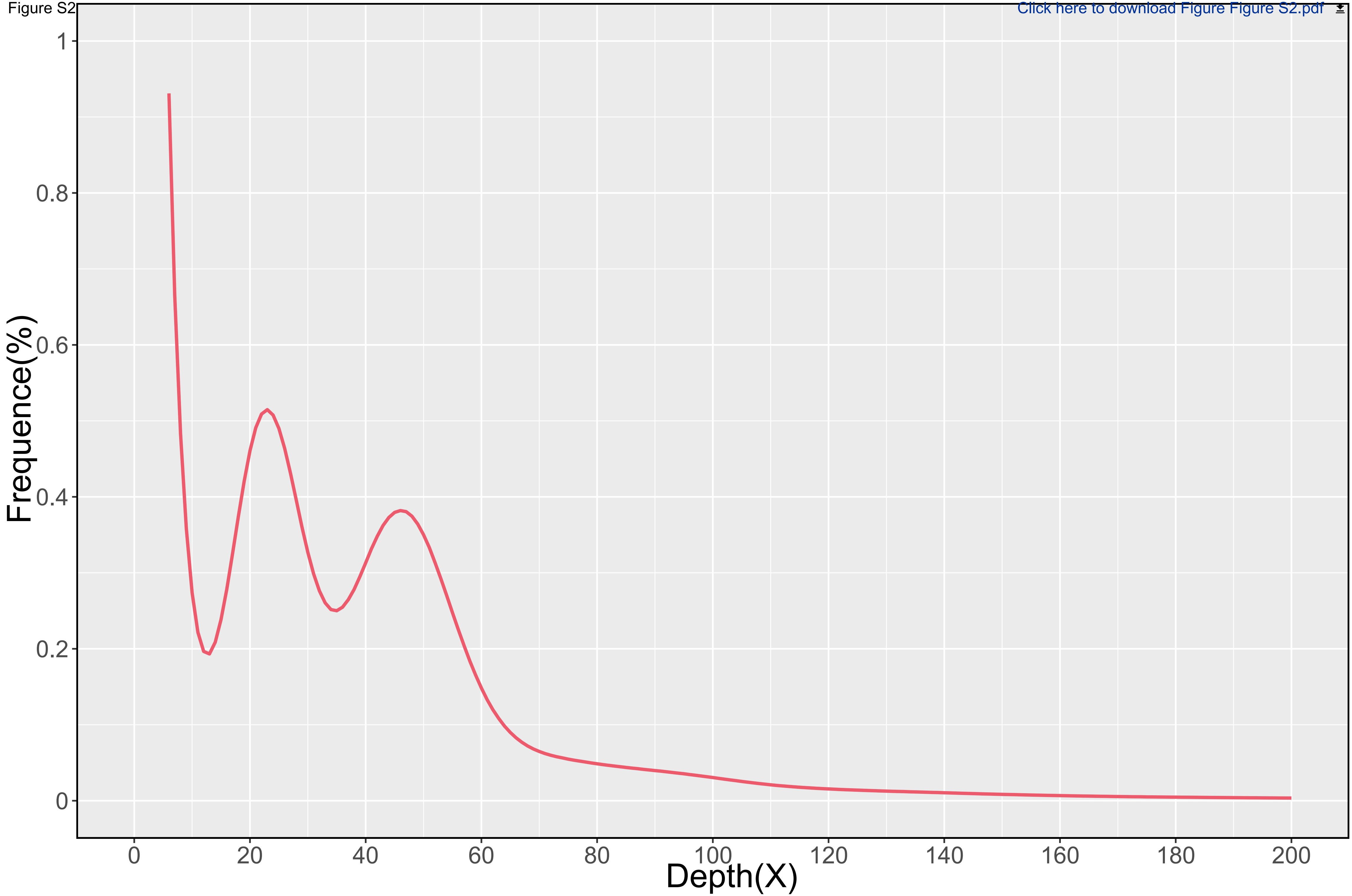

Click here to access/download
Supplementary Material
Additional File 1.docx

Dear Editors:

We would like to submit the enclosed manuscript entitled "Long-read sequence assembly of the firefly Pyrocoelia pectoralis genome", which we wish to be considered for publication as a Data Note in GigaScience. All authors have read and approved the final version of the manuscript and there is no competing interests need to be declared. We declare that this research is an original work and has not been submitted for other journals.

In this work, we developed a highly reliable genome resource for fireflies which are one of the most well known and loved insect species because of their bioluminescence using single molecule real time (SMRT) cells on the PacBio Sequel platform. In total, 57.8Gb long reads about 74X were generated and assembled into a final size of 760.7Mb genome which is close to the estimated genome size and covered 98.7% complete and 0.7% partial insect BUSCOs. Our long-read assembly demonstrates continuousness with a contig N50 length of 3.04Mb, which is the longest compared with other published insect genomes except for model insect Drosophila melanogaster, all the results suggested that the quality including base level accuracy and completeness of our assembly is very close to gold-standard reference genomes for fireflies. Furthermore, repeat sequencings and genes were also detected and gene functions were assigned. I hope this manuscript is suitable for Data Note in GigaScience, and those big data not only provide insights into conservation of fireflies

and biodiversity, but also provide a wealth information to study the mechanism of sexual communication, bio-luminescence and the evolution of insects.

We deeply appreciate your consideration of our manuscript, and look forward to hearing from the reviewers.

Sincerely yours,

Jiang Hu

Corresponding author:

Name: Jiang Hu

E-mail: huj@grandomics.com
