# Supplementary material for: Long-read sequence assembly of the firefly Pyrocoelia pectoralis genome
Source: Gigascience. 2017 Nov 24;6(12):1–7. doi: 10.1093/gigascience/gix112 (PMC5751067; doi:10.1093/gigascience/gix112)
Supplement: Supplemental material [file gix112_supp.zip › Additional File 1.docx]

### Supplementary Text

**MAKER command used for gene annotation**

blast_type=ncbi+

pcov_blastn=0.8

pid_blastn=0.85

eval_blastn=1e-10

bit_blastn=40

depth_blastn=0

pcov_blastx=0.5

pid_blastx=0.4

eval_blastx=1e-06

bit_blastx=30

depth_blastx=0

pcov_tblastx=0.8

pid_tblastx=0.85

eval_tblastx=1e-10

bit_tblastx=40

depth_tblastx=0

pcov_rm_blastx=0.5

pid_rm_blastx=0.4

eval_rm_blastx=1e-06

bit_rm_blastx=30

ep_score_limit=20

en_score_limit=20

genome=YHC.genome.fasta

organism_type=eukaryotic

est_pass=0

altest_pass=0

protein_pass=0

rm_pass=0

model_pass=0

pred_pass=0

other_pass=0

est=YHC.Denovo.Unigene.fasta,YHC.SRR4045940.Trinity.fasta

est_gff=AdvanceloSkins-1.transcripts.gff3,AdvanceloSkins-2.transcripts.gff3, Insectlo-1.transcripts.gff3, Insectlo-2.transcripts.gff3,MidAdvanceLo-1.transcripts.gff3,MidAdvanceLo-2.transcripts.gff3,MidLatelo-1.transcripts.gff3,MidLatelo-2.transcripts.gff3

protein=6Spp.protein.fasta

rm_gff=YHC.genome.fasta.repeatAll.rename.gff

prok_rm=0

softmask=1

snaphmm=YHC.hmm

gmhmm=ES_C.mod

augustus_species=YHC_0712

est2genome=0

protein2genome=0

trna=0

unmask=0

alt_peptide=C

cpus=1

max_dna_len=1000000

min_contig=10000

pred_flank=1000

pred_stats=1

AED_threshold=1

min_protein=0

alt_splice=0

always_complete=1

map_forward=0

keep_preds=0

split_hit=10000

single_exon=0

single_length=250

correct_est_fusion=0

tries=3

clean_try=0

clean_up=0

TMP=/tmp

### Supplementary Figures and Tables


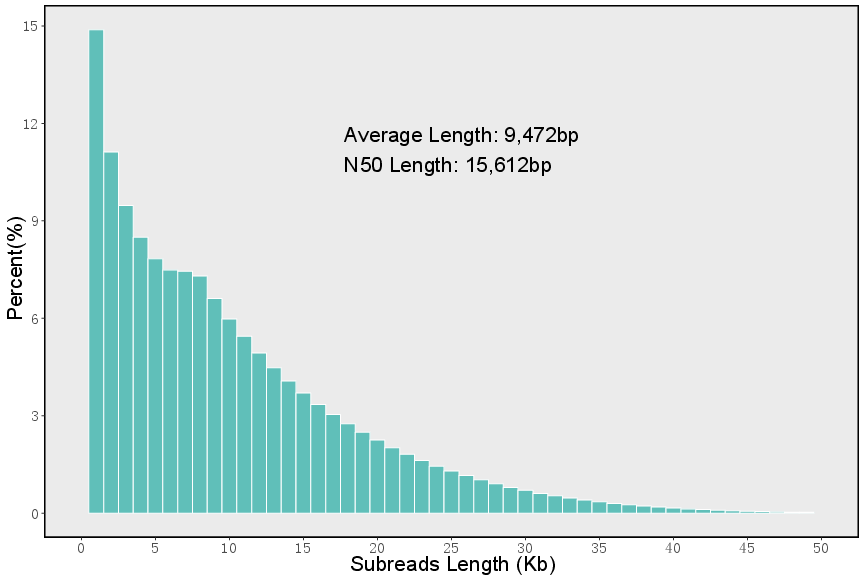


Figure S1: The length distribution of subreads. The read length of post-filter subreads from PacBio Sequel system that used to do assembly and the length of the longest read is 104,415bp (not shown).


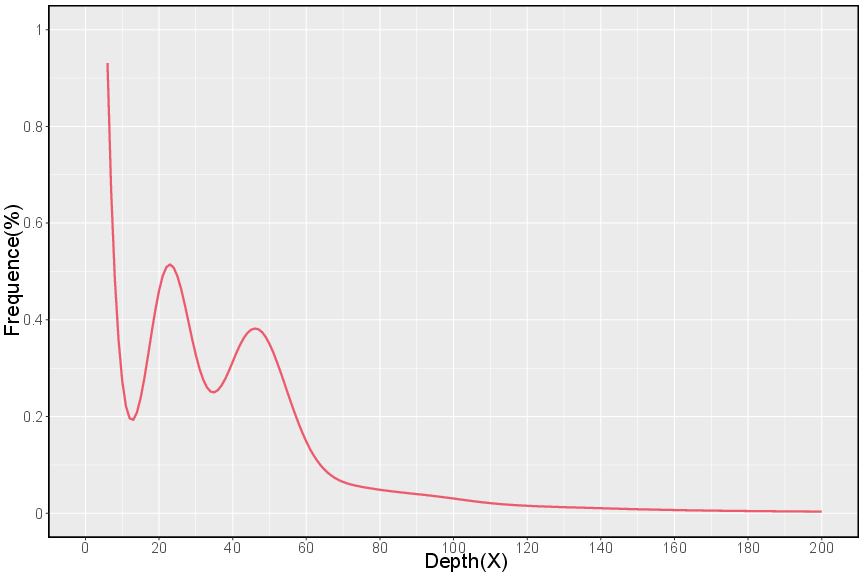


Figure S2: 17-mer depth distribution. The first peak (depth=23) is a heterozygous peak, which is higher than the main peak (depth=46), suggesting the firefly genome is a highly heterozygous genome.


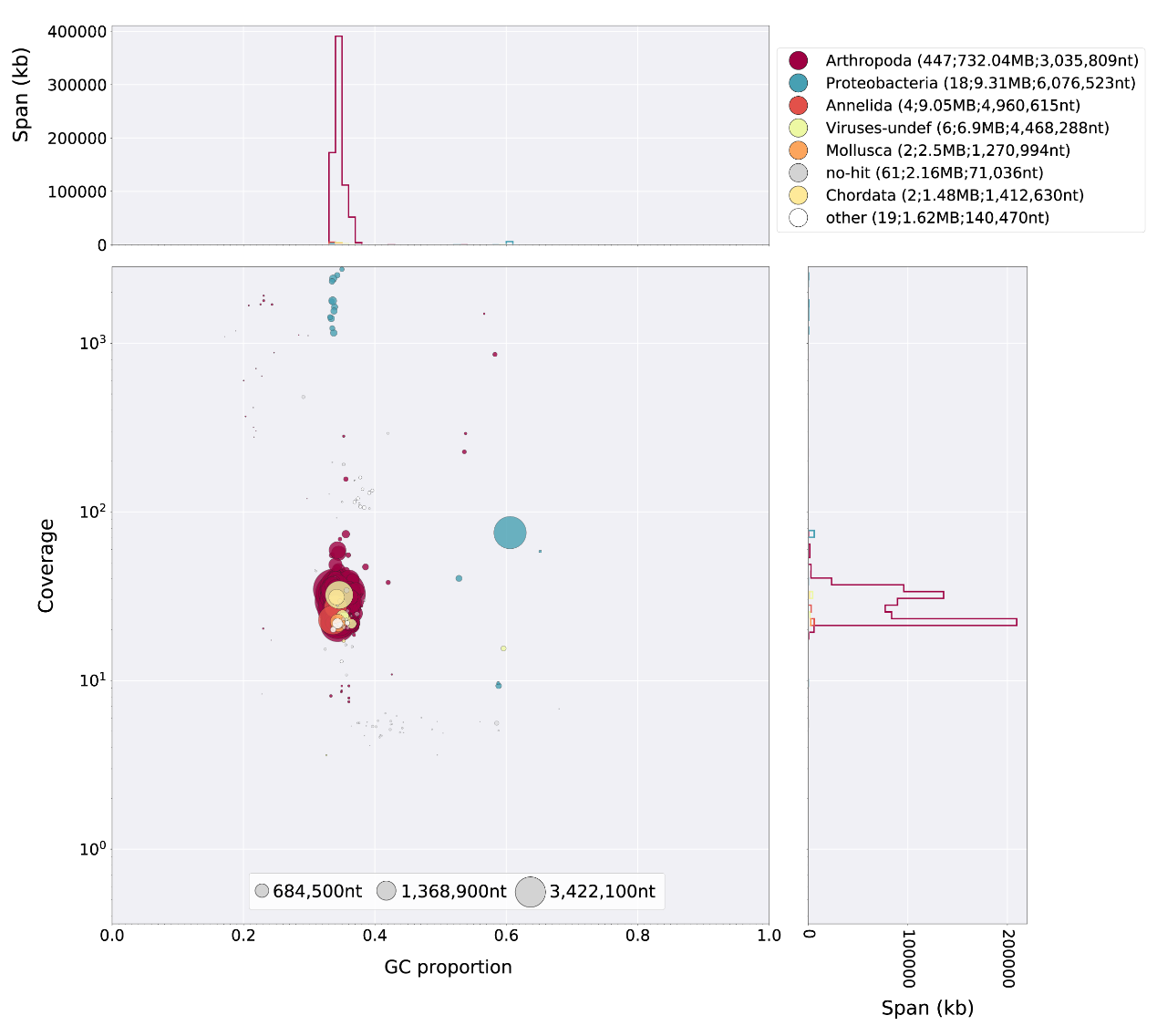


Figure S3: Taxon-annotated GC-coverage plots for final assembly. Each contig in the assembly is represented by a circle and coloured according to the best match to taxonomic annotation. The upper- and right-hand panels show the distribution of the total span (kb) of contigs for a given coverage (right panel) or GC (upper panel) bin.

Table S1: The statistics of sequencing data and library information.

| Library Type | Platform | Reads Number | Bases(bp) | Insert Size | Average Reads Length(bp) |
| --- | --- | --- | --- | --- | --- |
| Short Insert | Illumina x10 | 315,768,660 | 47,365,299,000 | 400 | 150 |
| Long Insert* | PacBio Sequel | 5,117,766 | 57,753,343,834 | 20,000 | 11,285 |

Note: * The statistics for PacBio Sequel platform are basing on raw reads (polymerase reads).

Table S2: Assessment of the completeness of the *P. pectoralis* genome using transcriptomic reads.

| Dataset | Total Reads | Pair Mapped | | Singleton Mapped | | Totally Mapped | |
| --- | --- | --- | --- | --- | --- | --- | --- |
|  |  | Number | Percent | Number | Percent | Number | Percent |
| Original assembly | 56,642,262 | 49,848,048 | 88.01% | 964,119 | 1.70% | 50,812,167 | 89.71% |
| Filtered assembly | 56,642,262 | 49,580,214 | 87.53% | 1,108,714 | 1.96% | 50,688,928 | 89.49% |

Table S3: The classification statistics of SSRs.

| Type | Unit size (repeat number) | Number | Percent | Most repeat unit | Most SSR |
| --- | --- | --- | --- | --- | --- |
| p1 | 1(>=10) | 95,886 | 70.72% | A | (A)_10_ |
| p2 | 2(>=6) | 7,947 | 5.86% | TA | (TA)_6_ |
| p3 | 3(>=5) | 26,748 | 19.73% | TAT | (AAT)_5_ |
| p4 | 4(>=5) | 3,105 | 2.29% | TTTA | (AATA)_5_ |
| p5 | 5(>=5) | 1,563 | 1.15% | TAAAC | (TAAAC)_5_ |
| p6 | 6(>=5) | 340 | 0.25% | CTTGAA | (AATATG)_5_ |
| Total | - | 135,589 | 100.00% | A | (A)_10_ |

Table S4: The statistics of final assembly.

| ID | Length | GC | Depth | Phylum.t.6 | Discarded |
| --- | --- | --- | --- | --- | --- |
| scaffold1 | 821085 | 0.3594 | 23.216 | Arthropoda | - |
| scaffold2 | 659174 | 0.3402 | 23.665 | Arthropoda | - |
| scaffold3 | 272584 | 0.3423 | 46.389 | Arthropoda | - |
| scaffold4 | 4636028 | 0.3477 | 22.148 | Arthropoda | - |
| scaffold5 | 2077307 | 0.3622 | 22.139 | Arthropoda | - |
| scaffold6 | 1568556 | 0.3504 | 22.036 | Arthropoda | - |
| scaffold7 | 1574584 | 0.3412 | 35.473 | Arthropoda | - |
| scaffold8 | 1572693 | 0.3452 | 29.178 | Arthropoda | - |
| scaffold9 | 1542867 | 0.3542 | 32.811 | Arthropoda | - |
| scaffold10 | 1549536 | 0.3362 | 29.609 | Arthropoda | - |
| scaffold11 | 1547580 | 0.3711 | 34.081 | Arthropoda | - |
| scaffold12 | 1526174 | 0.3408 | 33.518 | Arthropoda | - |
| scaffold13 | 71036 | 0.3586 | 20.575 | no-hit | - |
| scaffold14 | 1494382 | 0.3583 | 22.49 | Arthropoda | - |
| scaffold15 | 1492293 | 0.3443 | 21.743 | Arthropoda | - |
| scaffold16 | 1486156 | 0.3539 | 22.454 | Arthropoda | - |
| scaffold17 | 1454961 | 0.3473 | 21.709 | Arthropoda | - |
| scaffold18 | 1466511 | 0.366 | 33.831 | Arthropoda | - |
| scaffold19 | 1447001 | 0.34 | 39.847 | Arthropoda | - |
| scaffold20 | 1436578 | 0.3435 | 25.214 | Arthropoda | - |
| scaffold21 | 1440417 | 0.361 | 21.925 | Arthropoda | - |
| scaffold22 | 1402321 | 0.3431 | 21.479 | Arthropoda | - |
| scaffold23 | 1416342 | 0.335 | 37.009 | Arthropoda | - |
| scaffold24 | 1412630 | 0.3417 | 31.248 | Chordata | - |
| scaffold25 | 1379882 | 0.3366 | 22 | Arthropoda | - |
| scaffold26 | 1377538 | 0.3351 | 27.299 | Arthropoda | - |
| scaffold27 | 1387709 | 0.3628 | 31.243 | Arthropoda | - |
| scaffold28 | 1395796 | 0.3459 | 22.721 | Arthropoda | - |
| scaffold29 | 1374588 | 0.3568 | 22.343 | Arthropoda | - |
| scaffold30 | 1355255 | 0.3446 | 30.774 | Arthropoda | - |
| scaffold31 | 1345348 | 0.3505 | 22.22 | Arthropoda | - |
| scaffold32 | 1342439 | 0.3452 | 37.502 | Arthropoda | - |
| scaffold33 | 1334639 | 0.3545 | 30.565 | Arthropoda | - |
| scaffold34 | 1328417 | 0.3346 | 22.516 | Arthropoda | - |
| scaffold35 | 1294921 | 0.3354 | 23.141 | Arthropoda | - |
| scaffold36 | 1289616 | 0.3421 | 25.161 | Arthropoda | - |
| scaffold37 | 1299381 | 0.3358 | 36.497 | Arthropoda | - |
| scaffold38 | 1300243 | 0.3344 | 31.945 | Arthropoda | - |
| scaffold39 | 1289126 | 0.3651 | 21.156 | Arthropoda | - |
| scaffold40 | 1304663 | 0.3541 | 21.571 | Arthropoda | - |
| scaffold41 | 1275865 | 0.3468 | 30.69 | Arthropoda | - |
| scaffold42 | 1270994 | 0.3434 | 22.402 | Mollusca | - |
| scaffold43 | 1306255 | 0.3381 | 22.414 | Arthropoda | - |
| scaffold44 | 1269638 | 0.3375 | 22.405 | Arthropoda | - |
| scaffold45 | 1260724 | 0.3549 | 22.086 | Arthropoda | - |
| scaffold46 | 1266805 | 0.3434 | 22.584 | Arthropoda | - |
| scaffold47 | 1243654 | 0.3447 | 56.849 | Arthropoda | - |
| scaffold48 | 1233909 | 0.3441 | 21.508 | Mollusca | - |
| scaffold49 | 1218607 | 0.3355 | 21.753 | Arthropoda | - |
| scaffold50 | 1203812 | 0.345 | 23.551 | Arthropoda | - |
| scaffold51 | 1190308 | 0.346 | 36.754 | Arthropoda | - |
| scaffold52 | 1172287 | 0.3486 | 22.16 | Arthropoda | - |
| scaffold53 | 1193697 | 0.343 | 35.637 | Arthropoda | - |
| scaffold54 | 1182123 | 0.3607 | 33.297 | Arthropoda | - |
| scaffold55 | 1107233 | 0.3405 | 41.469 | Arthropoda | - |
| scaffold56 | 1115830 | 0.3369 | 33.486 | Arthropoda | - |
| scaffold57 | 1089406 | 0.3519 | 32.791 | Arthropoda | - |
| scaffold58 | 1082408 | 0.3604 | 32.11 | Arthropoda | - |
| scaffold59 | 1072782 | 0.3645 | 34.701 | Arthropoda | - |
| scaffold60 | 1073242 | 0.3365 | 21.878 | Arthropoda | - |
| scaffold61 | 1053957 | 0.34 | 48.593 | Arthropoda | - |
| scaffold62 | 1063096 | 0.364 | 34.565 | Arthropoda | - |
| scaffold63 | 1056620 | 0.3605 | 35.357 | Arthropoda | - |
| scaffold64 | 1023797 | 0.3375 | 31.664 | Arthropoda | - |
| scaffold65 | 1015923 | 0.3603 | 21.682 | Arthropoda | - |
| scaffold66 | 1000679 | 0.3576 | 22.894 | Arthropoda | - |
| scaffold67 | 1010421 | 0.3518 | 23.348 | Arthropoda | - |
| scaffold68 | 977629 | 0.3405 | 40.787 | Arthropoda | - |
| scaffold69 | 998673 | 0.3679 | 32.649 | Arthropoda | - |
| scaffold70 | 980864 | 0.3447 | 45.341 | Arthropoda | - |
| scaffold71 | 967056 | 0.3402 | 25.143 | Arthropoda | - |
| scaffold72 | 956855 | 0.3559 | 23.026 | Arthropoda | - |
| scaffold73 | 951722 | 0.344 | 21.533 | Arthropoda | - |
| scaffold74 | 943986 | 0.3585 | 26.518 | Arthropoda | - |
| scaffold75 | 961222 | 0.3463 | 31.363 | Arthropoda | - |
| scaffold76 | 934784 | 0.3617 | 33.596 | Arthropoda | - |
| scaffold77 | 939087 | 0.3456 | 21.244 | Arthropoda | - |
| scaffold78 | 931752 | 0.3401 | 22.389 | Arthropoda | - |
| scaffold79 | 915218 | 0.337 | 21.332 | Arthropoda | - |
| scaffold80 | 889085 | 0.3443 | 35.361 | Arthropoda | - |
| scaffold81 | 880184 | 0.3548 | 21.606 | Arthropoda | - |
| scaffold82 | 878397 | 0.3507 | 24.064 | Viruses-undef | - |
| scaffold83 | 891081 | 0.3655 | 22.461 | Arthropoda | - |
| scaffold84 | 883496 | 0.3597 | 33.187 | Arthropoda | - |
| scaffold85 | 863746 | 0.366 | 39.627 | Arthropoda | - |
| scaffold86 | 877567 | 0.3624 | 35.54 | Arthropoda | - |
| scaffold87 | 890062 | 0.3354 | 28.463 | Arthropoda | - |
| scaffold88 | 876327 | 0.3442 | 33.772 | Arthropoda | - |
| scaffold89 | 873328 | 0.3446 | 36.573 | Arthropoda | - |
| scaffold90 | 858810 | 0.3528 | 23.37 | Viruses-undef | - |
| scaffold91 | 866999 | 0.3448 | 24.572 | Arthropoda | - |
| scaffold92 | 854787 | 0.3581 | 31.27 | Arthropoda | - |
| scaffold93 | 825135 | 0.344 | 38.015 | Arthropoda | - |
| scaffold94 | 841079 | 0.3388 | 38.279 | Arthropoda | - |
| scaffold95 | 832476 | 0.3595 | 23.05 | Arthropoda | - |
| scaffold96 | 807904 | 0.3455 | 22.985 | Arthropoda | - |
| scaffold97 | 788038 | 0.34 | 21.393 | Arthropoda | - |
| scaffold98 | 818073 | 0.3512 | 37.228 | Arthropoda | - |
| scaffold99 | 810702 | 0.35 | 23.252 | Arthropoda | - |
| scaffold100 | 813338 | 0.3436 | 23.765 | Arthropoda | - |
| scaffold101 | 794206 | 0.3435 | 21.594 | Arthropoda | - |
| scaffold102 | 791813 | 0.3359 | 21.931 | Arthropoda | - |
| scaffold103 | 785235 | 0.3475 | 22.226 | Arthropoda | - |
| scaffold104 | 787006 | 0.3366 | 25.756 | Arthropoda | - |
| scaffold105 | 775942 | 0.3501 | 22.375 | Arthropoda | - |
| scaffold106 | 788013 | 0.3341 | 26.393 | Arthropoda | - |
| scaffold107 | 779620 | 0.3391 | 35.209 | Arthropoda | - |
| scaffold108 | 777601 | 0.3403 | 32.061 | Arthropoda | - |
| scaffold109 | 356953 | 0.3435 | 25.925 | Arthropoda | - |
| scaffold110 | 758100 | 0.3589 | 36.658 | Arthropoda | - |
| scaffold111 | 751317 | 0.3665 | 29.011 | Arthropoda | - |
| scaffold112 | 730204 | 0.3462 | 20.735 | Arthropoda | - |
| scaffold113 | 748017 | 0.3625 | 31.873 | Arthropoda | - |
| scaffold114 | 743107 | 0.35 | 32.723 | Arthropoda | - |
| scaffold115 | 744449 | 0.3515 | 26.816 | Arthropoda | - |
| scaffold116 | 714909 | 0.3446 | 21.491 | Arthropoda | - |
| scaffold117 | 727681 | 0.3525 | 35.783 | Arthropoda | - |
| scaffold118 | 723389 | 0.3393 | 33.698 | Arthropoda | - |
| scaffold119 | 726429 | 0.3623 | 21.689 | Arthropoda | - |
| scaffold120 | 605972 | 0.3464 | 21.427 | Arthropoda | - |
| scaffold121 | 719763 | 0.3432 | 35.387 | Arthropoda | - |
| scaffold122 | 736793 | 0.3647 | 35.711 | Arthropoda | - |
| scaffold123 | 717394 | 0.341 | 28.346 | Arthropoda | - |
| scaffold124 | 404666 | 0.3722 | 34.311 | Arthropoda | - |
| scaffold125 | 715672 | 0.3427 | 34.938 | Arthropoda | - |
| scaffold126 | 704285 | 0.3586 | 21.457 | Arthropoda | - |
| scaffold127 | 689923 | 0.3454 | 21.566 | Arthropoda | - |
| scaffold128 | 702504 | 0.3357 | 24.819 | Arthropoda | - |
| scaffold129 | 695690 | 0.3525 | 22.072 | Arthropoda | - |
| scaffold130 | 691435 | 0.3381 | 22.192 | Arthropoda | - |
| scaffold131 | 374521 | 0.3561 | 23.023 | Arthropoda | - |
| scaffold132 | 683459 | 0.3417 | 22.889 | Arthropoda | - |
| scaffold133 | 677898 | 0.3469 | 37.416 | Arthropoda | - |
| scaffold134 | 683848 | 0.345 | 26.696 | Arthropoda | - |
| scaffold135 | 660144 | 0.3578 | 27.161 | Arthropoda | - |
| scaffold136 | 663730 | 0.3715 | 31.526 | Arthropoda | - |
| scaffold137 | 661939 | 0.3579 | 25.654 | Arthropoda | - |
| scaffold138 | 652875 | 0.3464 | 24.704 | Arthropoda | - |
| scaffold139 | 650591 | 0.3584 | 29.591 | Arthropoda | - |
| scaffold140 | 629990 | 0.3459 | 40.338 | Arthropoda | - |
| scaffold141 | 644226 | 0.3416 | 24.398 | Arthropoda | - |
| scaffold142 | 621349 | 0.3492 | 22.22 | Arthropoda | - |
| scaffold143 | 636749 | 0.3562 | 22.171 | Arthropoda | - |
| scaffold144 | 626579 | 0.3585 | 21.638 | Arthropoda | - |
| scaffold145 | 628118 | 0.3664 | 20.77 | Arthropoda | - |
| scaffold146 | 603015 | 0.3539 | 26.729 | Arthropoda | - |
| scaffold147 | 612786 | 0.3572 | 22.701 | Annelida | - |
| scaffold148 | 638910 | 0.3585 | 29.185 | Arthropoda | - |
| scaffold149 | 613622 | 0.3702 | 34.006 | Arthropoda | - |
| scaffold150 | 606773 | 0.3436 | 21.401 | Arthropoda | - |
| scaffold151 | 602772 | 0.3566 | 22.34 | Arthropoda | - |
| scaffold152 | 608826 | 0.3431 | 21.785 | Nematoda | - |
| scaffold153 | 618151 | 0.3468 | 29.542 | Arthropoda | - |
| scaffold154 | 602857 | 0.3442 | 21.701 | Arthropoda | - |
| scaffold155 | 492902 | 0.3522 | 33.877 | Arthropoda | - |
| scaffold156 | 592861 | 0.342 | 23.643 | Arthropoda | - |
| scaffold157 | 569554 | 0.3475 | 23.093 | Arthropoda | - |
| scaffold158 | 581962 | 0.3478 | 21.156 | Arthropoda | - |
| scaffold159 | 586632 | 0.342 | 23.535 | Arthropoda | - |
| scaffold160 | 585489 | 0.3394 | 38.902 | Arthropoda | - |
| scaffold161 | 581509 | 0.3389 | 24.86 | Arthropoda | - |
| scaffold162 | 556068 | 0.344 | 21.476 | Arthropoda | - |
| scaffold163 | 564928 | 0.3619 | 22.209 | Arthropoda | - |
| scaffold164 | 555776 | 0.3522 | 25.384 | Arthropoda | - |
| scaffold165 | 541080 | 0.3393 | 21.85 | Arthropoda | - |
| scaffold166 | 540989 | 0.3313 | 22.066 | Arthropoda | - |
| scaffold167 | 527698 | 0.3451 | 21.933 | Arthropoda | - |
| scaffold168 | 496725 | 0.3565 | 25.852 | Arthropoda | - |
| scaffold169 | 527477 | 0.3418 | 21.274 | Arthropoda | - |
| scaffold170 | 512844 | 0.3431 | 22.079 | Annelida | - |
| scaffold171 | 524825 | 0.3646 | 21.716 | Viruses-undef | - |
| scaffold172 | 519722 | 0.3523 | 22.71 | Arthropoda | - |
| scaffold173 | 508644 | 0.3313 | 21.406 | Arthropoda | - |
| scaffold174 | 499601 | 0.3364 | 24.08 | Arthropoda | - |
| scaffold175 | 489743 | 0.3441 | 23.525 | Arthropoda | - |
| scaffold176 | 491801 | 0.3656 | 20.956 | Arthropoda | - |
| scaffold177 | 488458 | 0.3593 | 35.792 | Arthropoda | - |
| scaffold178 | 482203 | 0.3405 | 32.045 | Arthropoda | - |
| scaffold179 | 201868 | 0.3358 | 28.09 | Arthropoda | - |
| scaffold180 | 480524 | 0.3636 | 22.805 | Arthropoda | - |
| scaffold181 | 471377 | 0.3428 | 22.495 | Arthropoda | - |
| scaffold182 | 462419 | 0.3644 | 21.724 | Arthropoda | - |
| scaffold183 | 455544 | 0.3404 | 23.646 | Arthropoda | - |
| scaffold184 | 467163 | 0.3655 | 31.374 | Arthropoda | - |
| scaffold185 | 434399 | 0.3551 | 27.551 | Arthropoda | - |
| scaffold186 | 417095 | 0.3384 | 34.205 | Arthropoda | - |
| scaffold187 | 415286 | 0.37 | 21.428 | Arthropoda | - |
| scaffold188 | 415527 | 0.347 | 20.224 | Arthropoda | - |
| scaffold189 | 404993 | 0.3413 | 43.458 | Arthropoda | - |
| scaffold190 | 403638 | 0.3456 | 22.236 | Arthropoda | - |
| scaffold191 | 399932 | 0.3622 | 24.963 | Arthropoda | - |
| scaffold192 | 387161 | 0.3371 | 23.043 | Arthropoda | - |
| scaffold193 | 389238 | 0.3537 | 22.132 | Arthropoda | - |
| scaffold194 | 386329 | 0.3362 | 22.345 | Arthropoda | - |
| scaffold195 | 377456 | 0.3373 | 22.358 | Arthropoda | - |
| scaffold196 | 365037 | 0.3403 | 28.258 | Arthropoda | - |
| scaffold197 | 372933 | 0.3688 | 21.154 | Arthropoda | - |
| scaffold198 | 370515 | 0.3481 | 31.426 | Arthropoda | - |
| scaffold199 | 369723 | 0.3624 | 33.201 | Arthropoda | - |
| scaffold200 | 365732 | 0.3579 | 29.643 | Arthropoda | - |
| scaffold201 | 377431 | 0.342 | 22.469 | Arthropoda | - |
| scaffold202 | 360668 | 0.3428 | 21.846 | Arthropoda | - |
| scaffold203 | 357705 | 0.3465 | 22.537 | Arthropoda | - |
| scaffold204 | 351436 | 0.3495 | 38.887 | Arthropoda | - |
| scaffold205 | 338712 | 0.3558 | 44.673 | Arthropoda | - |
| scaffold206 | 352678 | 0.3558 | 73.839 | Arthropoda | - |
| scaffold207 | 353263 | 0.3689 | 22.008 | Arthropoda | - |
| scaffold208 | 348031 | 0.3695 | 30.525 | Arthropoda | - |
| scaffold209 | 318411 | 0.3576 | 20.268 | Arthropoda | - |
| scaffold210 | 319130 | 0.3509 | 27.714 | Arthropoda | - |
| scaffold211 | 313840 | 0.341 | 25.499 | Arthropoda | - |
| scaffold212 | 298737 | 0.3528 | 35.202 | Arthropoda | - |
| scaffold213 | 302793 | 0.3471 | 34.189 | Arthropoda | - |
| scaffold214 | 302147 | 0.3473 | 34.535 | Arthropoda | - |
| scaffold215 | 275953 | 0.3472 | 43.556 | Arthropoda | - |
| scaffold216 | 291823 | 0.3423 | 22.597 | Arthropoda | - |
| scaffold217 | 246271 | 0.3674 | 33.046 | Arthropoda | - |
| scaffold218 | 259761 | 0.3513 | 22.56 | Arthropoda | - |
| scaffold219 | 252397 | 0.3457 | 22.207 | Arthropoda | - |
| scaffold220 | 227892 | 0.3857 | 47.172 | Arthropoda | - |
| scaffold221 | 223365 | 0.3551 | 19.344 | Arthropoda | - |
| scaffold222 | 225328 | 0.3394 | 35.34 | Arthropoda | - |
| scaffold223 | 218635 | 0.3551 | 19.33 | Arthropoda | - |
| scaffold224 | 217993 | 0.3557 | 21.582 | Arthropoda | - |
| scaffold225 | 212004 | 0.3383 | 26.944 | Arthropoda | - |
| scaffold226 | 201608 | 0.3536 | 21.148 | Arthropoda | - |
| scaffold227 | 199166 | 0.3433 | 21.221 | Arthropoda | - |
| scaffold228 | 196548 | 0.3432 | 33.922 | Arthropoda | - |
| scaffold229 | 195003 | 0.3365 | 20.04 | Nematoda | - |
| scaffold230 | 194042 | 0.3362 | 21.88 | Arthropoda | - |
| scaffold231 | 187774 | 0.3534 | 21.593 | Arthropoda | - |
| scaffold232 | 186451 | 0.3684 | 28.027 | Arthropoda | - |
| scaffold233 | 164282 | 0.3426 | 22.567 | Arthropoda | - |
| scaffold234 | 13688299 | 0.3486 | 32.627 | Arthropoda | - |
| scaffold235 | 10393053 | 0.3416 | 32.447 | Arthropoda | - |
| scaffold236 | 9647767 | 0.3398 | 29.546 | Arthropoda | - |
| scaffold237 | 9637660 | 0.3372 | 34.74 | Arthropoda | - |
| scaffold238 | 9125646 | 0.3451 | 31.825 | Arthropoda | - |
| scaffold239 | 7335335 | 0.3499 | 32.43 | Arthropoda | - |
| scaffold240 | 7290401 | 0.3437 | 33.529 | Arthropoda | - |
| scaffold241 | 6710636 | 0.3433 | 21.412 | Arthropoda | - |
| scaffold242 | 6335920 | 0.3398 | 32.96 | Arthropoda | - |
| scaffold243 | 177899 | 0.3346 | 55.326 | Arthropoda | - |
| scaffold244 | 173261 | 0.3607 | 23.928 | Arthropoda | - |
| scaffold245 | 159418 | 0.3486 | 21.573 | no-hit | - |
| scaffold246 | 164283 | 0.3569 | 34.28 | no-hit | - |
| scaffold247 | 158325 | 0.3328 | 20.375 | Arthropoda | - |
| scaffold248 | 161018 | 0.3409 | 23.466 | Arthropoda | - |
| scaffold249 | 159710 | 0.344 | 28.158 | Arthropoda | - |
| scaffold250 | 153505 | 0.3373 | 20.149 | Arthropoda | - |
| scaffold251 | 150681 | 0.3594 | 55.395 | Arthropoda | - |
| scaffold252 | 150208 | 0.3437 | 22.34 | Arthropoda | - |
| scaffold253 | 147002 | 0.3615 | 21.205 | Arthropoda | - |
| scaffold254 | 140470 | 0.358 | 21.915 | undef | - |
| scaffold255 | 133607 | 0.3558 | 156.411 | Arthropoda | - |
| scaffold256 | 122864 | 0.3727 | 24.821 | no-hit | - |
| scaffold257 | 120111 | 0.3451 | 22.81 | Arthropoda | - |
| scaffold258 | 119721 | 0.3442 | 33.682 | Arthropoda | - |
| scaffold259 | 119300 | 0.4202 | 38.175 | Arthropoda | - |
| scaffold260 | 115707 | 0.3469 | 29.65 | no-hit | - |
| scaffold261 | 114435 | 0.5827 | 858.47 | Arthropoda | - |
| scaffold262 | 117344 | 0.3431 | 19.115 | Arthropoda | - |
| scaffold263 | 112309 | 0.3468 | 22.507 | Arthropoda | - |
| scaffold264 | 109316 | 0.3452 | 21.515 | no-hit | - |
| scaffold265 | 102275 | 0.5361 | 227.023 | Arthropoda | - |
| scaffold266 | 101565 | 0.3398 | 21.184 | no-hit | - |
| scaffold267 | 90136 | 0.3469 | 68.977 | Arthropoda | - |
| scaffold268 | 88537 | 0.3643 | 31.258 | Arthropoda | - |
| scaffold269 | 85333 | 0.3414 | 28.154 | Arthropoda | - |
| scaffold270 | 83504 | 0.3531 | 20.668 | no-hit | - |
| scaffold271 | 84345 | 0.3303 | 20.475 | Arthropoda | - |
| scaffold272 | 75890 | 0.3567 | 22.964 | no-hit | - |
| scaffold273 | 75714 | 0.3742 | 21.438 | Arthropoda | - |
| scaffold274 | 71577 | 0.3682 | 18.662 | Arthropoda | - |
| scaffold275 | 69410 | 0.3755 | 24.173 | Arthropoda | - |
| scaffold276 | 68651 | 0.3584 | 25.839 | no-hit | - |
| scaffold277 | 68302 | 0.3461 | 20.636 | Arthropoda | - |
| scaffold278 | 68158 | 0.2911 | 480.173 | no-hit | - |
| scaffold279 | 66975 | 0.3465 | 21.184 | no-hit | - |
| scaffold280 | 66770 | 0.3269 | 22.661 | Arthropoda | - |
| scaffold281 | 65570 | 0.3519 | 23.154 | no-hit | - |
| scaffold282 | 64496 | 0.3525 | 17.176 | Chordata | - |
| scaffold283 | 57440 | 0.3796 | 27.762 | Arthropoda | - |
| scaffold284 | 56209 | 0.3493 | 12.976 | Cnidaria | - |
| scaffold285 | 54950 | 0.3524 | 191.159 | no-hit | - |
| scaffold286 | 51061 | 0.333 | 8.104 | Arthropoda | - |
| scaffold287 | 45170 | 0.3747 | 23.54 | Arthropoda | - |
| scaffold288 | 42659 | 0.3525 | 281.004 | Arthropoda | - |
| scaffold289 | 41857 | 0.339 | 20.428 | no-hit | - |
| scaffold290 | 40810 | 0.384 | 29.955 | no-hit | - |
| scaffold291 | 39713 | 0.3965 | 5.344 | no-hit | - |
| scaffold292 | 33612 | 0.4232 | 5.123 | no-hit | Discarded |
| scaffold293 | 37764 | 0.3654 | 15.874 | no-hit | - |
| scaffold294 | 37566 | 0.3605 | 9.294 | Arthropoda | - |
| scaffold295 | 37555 | 0.3604 | 7.486 | Arthropoda | - |
| scaffold296 | 37544 | 0.3605 | 7.879 | Arthropoda | - |
| scaffold297 | 35017 | 0.3432 | 27.75 | no-hit | - |
| scaffold298 | 34433 | 0.3557 | 16.275 | no-hit | - |
| scaffold299 | 33885 | 0.3399 | 23.038 | no-hit | - |
| scaffold300 | 30681 | 0.4016 | 5.326 | no-hit | Discarded |
| scaffold301 | 28763 | 0.3642 | 24.161 | no-hit | - |
| scaffold302 | 27483 | 0.3797 | 32.416 | Arthropoda | - |
| scaffold303 | 26291 | 0.3489 | 8.559 | Arthropoda | - |
| scaffold304 | 26093 | 0.3493 | 8.696 | Arthropoda | - |
| scaffold305 | 26036 | 0.3496 | 9.282 | Arthropoda | - |
| scaffold306 | 23356 | 0.4241 | 5.75 | no-hit | Discarded |
| scaffold307 | 22512 | 0.5663 | 1496.882 | Arthropoda | - |
| scaffold308 | 21171 | 0.4417 | 5.227 | no-hit | Discarded |
| scaffold309 | 20280 | 0.374 | 5.608 | no-hit | Discarded |
| scaffold310 | 18352 | 0.4072 | 4.613 | no-hit | Discarded |
| scaffold311 | 18147 | 0.3763 | 5.609 | no-hit | Discarded |
| scaffold312 | 17698 | 0.3383 | 21.64 | no-hit | - |
| scaffold313 | 17396 | 0.4258 | 10.874 | Arthropoda | - |
| scaffold314 | 16767 | 0.4047 | 5.776 | no-hit | Discarded |
| scaffold315 | 15080 | 0.4382 | 4.917 | no-hit | Discarded |
| scaffold316 | 14881 | 0.5881 | 5.043 | no-hit | Discarded |
| scaffold317 | 14156 | 0.3714 | 118.923 | no-hit | - |
| scaffold318 | 12945 | 0.4159 | 6.394 | no-hit | Discarded |
| scaffold319 | 11338 | 0.4104 | 4.716 | no-hit | Discarded |
| scaffold320 | 10081 | 0.487 | 5.137 | no-hit | Discarded |
| scaffold321 | 10041 | 0.4242 | 5.489 | no-hit | Discarded |
| scaffold322 | 9920 | 0.4085 | 4.748 | no-hit | - |
| scaffold323 | 8203 | 0.4262 | 5.511 | no-hit | Discarded |
| scaffold324 | 7810 | 0.3352 | 196.787 | no-hit | - |
| scaffold325 | 7572 | 0.3722 | 117.738 | no-hit | - |
| scaffold326 | 7044 | 0.4708 | 6.027 | no-hit | Discarded |
| scaffold327 | 6787 | 0.4432 | 5.659 | no-hit | - |
| scaffold328 | 6443 | 0.433 | 6.168 | no-hit | Discarded |
| scaffold329 | 5612 | 0.3867 | 5.389 | no-hit | Discarded |
| scaffold330 | 5203 | 0.388 | 5.679 | no-hit | Discarded |
| scaffold331 | 4699 | 0.4948 | 3.618 | no-hit | Discarded |
| scaffold332 | 4495 | 0.4436 | 4.901 | no-hit | Discarded |
| scaffold333 | 4185 | 0.3391 | 41.136 | no-hit | - |
| scaffold334 | 4029 | 0.3842 | 4.71 | no-hit | Discarded |
| scaffold335 | 3450 | 0.3643 | 5.358 | no-hit | Discarded |
| scaffold336 | 3008 | 0.3088 | 45.521 | no-hit | - |
| scaffold337 | 2755 | 0.5601 | 5.693 | no-hit | Discarded |
| scaffold338 | 3759 | 0.3418 | 92.313 | no-hit | - |
| scaffold339 | 2734 | 0.5037 | 4.879 | no-hit | Discarded |
| scaffold340 | 2591 | 0.3921 | 4.119 | no-hit | Discarded |
| scaffold341 | 2547 | 0.4947 | 5.712 | no-hit | Discarded |
| scaffold342 | 46607 | 0.538 | 291.64 | Arthropoda | - |
| scaffold343 | 6283869 | 0.3477 | 29.981 | Arthropoda | - |
| scaffold344 | 6076523 | 0.6057 | 75.329 | Proteobacteria | - |
| scaffold345 | 6090314 | 0.3434 | 22.031 | Arthropoda | - |
| scaffold346 | 5947523 | 0.3503 | 25.514 | Arthropoda | - |
| scaffold347 | 5880183 | 0.345 | 31.318 | Arthropoda | - |
| scaffold348 | 5728231 | 0.3482 | 22.138 | Arthropoda | - |
| scaffold349 | 5500403 | 0.3414 | 23.32 | Arthropoda | - |
| scaffold350 | 5527040 | 0.3471 | 29.109 | Arthropoda | - |
| scaffold351 | 5428064 | 0.3492 | 26.125 | Arthropoda | - |
| scaffold352 | 5336451 | 0.3401 | 33.937 | Arthropoda | - |
| scaffold353 | 5266341 | 0.3373 | 28.239 | Arthropoda | - |
| scaffold354 | 5254721 | 0.3398 | 22.175 | Arthropoda | - |
| scaffold355 | 5185579 | 0.3355 | 26.348 | Arthropoda | - |
| scaffold356 | 5233057 | 0.3484 | 26.287 | Arthropoda | - |
| scaffold357 | 5085789 | 0.3408 | 33.668 | Arthropoda | - |
| scaffold358 | 5043425 | 0.3444 | 29.801 | Arthropoda | - |
| scaffold359 | 5008774 | 0.3516 | 22.841 | Arthropoda | - |
| scaffold360 | 4995932 | 0.3397 | 36.726 | Arthropoda | - |
| scaffold361 | 4960615 | 0.3363 | 22.903 | Annelida | - |
| scaffold362 | 4933506 | 0.3452 | 32.967 | Arthropoda | - |
| scaffold363 | 4888130 | 0.3382 | 27.592 | Arthropoda | - |
| scaffold364 | 4801893 | 0.3421 | 25.189 | Arthropoda | - |
| scaffold365 | 4773218 | 0.3409 | 25.054 | Arthropoda | - |
| scaffold366 | 4779667 | 0.352 | 34.135 | Arthropoda | - |
| scaffold367 | 4753360 | 0.3412 | 35.732 | Arthropoda | - |
| scaffold368 | 4758491 | 0.3395 | 22.554 | Arthropoda | - |
| scaffold369 | 4563139 | 0.3481 | 23.497 | Arthropoda | - |
| scaffold370 | 4535286 | 0.3386 | 25.481 | Arthropoda | - |
| scaffold371 | 4468288 | 0.3456 | 32.191 | Viruses-undef | - |
| scaffold372 | 4036292 | 0.3415 | 28.34 | Arthropoda | - |
| scaffold373 | 4354097 | 0.3381 | 34.94 | Arthropoda | - |
| scaffold374 | 4360506 | 0.3457 | 21.978 | Arthropoda | - |
| scaffold375 | 4350119 | 0.339 | 24.936 | Arthropoda | - |
| scaffold376 | 4309286 | 0.3389 | 32.695 | Arthropoda | - |
| scaffold377 | 4316926 | 0.3437 | 38.854 | Arthropoda | - |
| scaffold378 | 4185421 | 0.339 | 29.332 | Arthropoda | - |
| scaffold379 | 4180658 | 0.3462 | 35.736 | Arthropoda | - |
| scaffold380 | 4177264 | 0.3407 | 24.25 | Arthropoda | - |
| scaffold381 | 4131718 | 0.3408 | 23.888 | Arthropoda | - |
| scaffold382 | 4151576 | 0.3409 | 27.38 | Arthropoda | - |
| scaffold383 | 4073153 | 0.3565 | 27.733 | Arthropoda | - |
| scaffold384 | 4020785 | 0.3412 | 32.473 | Arthropoda | - |
| scaffold385 | 3920046 | 0.3455 | 34.964 | Arthropoda | - |
| scaffold386 | 3899404 | 0.3364 | 31.15 | Arthropoda | - |
| scaffold387 | 3845369 | 0.3449 | 22.068 | Arthropoda | - |
| scaffold388 | 3896490 | 0.3379 | 32.406 | Arthropoda | - |
| scaffold389 | 3840501 | 0.3509 | 22.688 | Arthropoda | - |
| scaffold390 | 3734992 | 0.3536 | 29.942 | Arthropoda | - |
| scaffold391 | 3589144 | 0.3608 | 32.107 | Arthropoda | - |
| scaffold392 | 3548254 | 0.3398 | 23.174 | Arthropoda | - |
| scaffold393 | 3506631 | 0.3425 | 27.758 | Arthropoda | - |
| scaffold394 | 3481985 | 0.3442 | 32.121 | Arthropoda | - |
| scaffold395 | 3522536 | 0.3553 | 37.2 | Arthropoda | - |
| scaffold396 | 3468317 | 0.3399 | 25.65 | Arthropoda | - |
| scaffold397 | 3568768 | 0.3552 | 34.912 | Arthropoda | - |
| scaffold398 | 3423677 | 0.3421 | 22.605 | Arthropoda | - |
| scaffold399 | 3394405 | 0.36 | 26.802 | Arthropoda | - |
| scaffold400 | 3288010 | 0.3489 | 22.621 | Arthropoda | - |
| scaffold401 | 3263406 | 0.349 | 28.171 | Arthropoda | - |
| scaffold402 | 3249311 | 0.3413 | 22.933 | Arthropoda | - |
| scaffold403 | 3180765 | 0.3618 | 29.348 | Arthropoda | - |
| scaffold404 | 3158354 | 0.3474 | 21.672 | Arthropoda | - |
| scaffold405 | 3135453 | 0.3426 | 36.312 | Arthropoda | - |
| scaffold406 | 3112863 | 0.3361 | 34.369 | Arthropoda | - |
| scaffold407 | 3072174 | 0.3493 | 32.649 | Arthropoda | - |
| scaffold408 | 3120633 | 0.3364 | 32.548 | Arthropoda | - |
| scaffold409 | 3035809 | 0.3413 | 29.993 | Arthropoda | - |
| scaffold410 | 3038261 | 0.3495 | 21.623 | Arthropoda | - |
| scaffold411 | 3023150 | 0.3428 | 22.064 | Arthropoda | - |
| scaffold412 | 3036029 | 0.3413 | 35.208 | Arthropoda | - |
| scaffold413 | 3004919 | 0.3457 | 28.408 | Arthropoda | - |
| scaffold414 | 2978068 | 0.3603 | 35.889 | Arthropoda | - |
| scaffold415 | 2966629 | 0.3402 | 27.446 | Annelida | - |
| scaffold416 | 2889779 | 0.3369 | 22.464 | Arthropoda | - |
| scaffold417 | 2831650 | 0.3444 | 32.627 | Arthropoda | - |
| scaffold418 | 2916294 | 0.3515 | 26.884 | Arthropoda | - |
| scaffold419 | 2854450 | 0.3603 | 22.006 | Arthropoda | - |
| scaffold420 | 2804625 | 0.3385 | 22.105 | Arthropoda | - |
| scaffold421 | 2771427 | 0.3445 | 26.764 | Arthropoda | - |
| scaffold422 | 2747515 | 0.3362 | 22.289 | Arthropoda | - |
| scaffold423 | 2718730 | 0.3523 | 21.625 | Arthropoda | - |
| scaffold424 | 2720187 | 0.3404 | 23.229 | Arthropoda | - |
| scaffold425 | 2745115 | 0.3428 | 34.956 | Arthropoda | - |
| scaffold426 | 2672986 | 0.3579 | 23.188 | Arthropoda | - |
| scaffold427 | 2750820 | 0.3503 | 29.172 | Arthropoda | - |
| scaffold428 | 2702450 | 0.3425 | 27.203 | Arthropoda | - |
| scaffold429 | 2671906 | 0.3433 | 21.808 | Arthropoda | - |
| scaffold430 | 2620084 | 0.3486 | 34.454 | Arthropoda | - |
| scaffold431 | 1559175 | 0.342 | 24.811 | Arthropoda | - |
| scaffold432 | 2638388 | 0.3558 | 29.462 | Arthropoda | - |
| scaffold433 | 2607585 | 0.3372 | 24.404 | Arthropoda | - |
| scaffold434 | 2595723 | 0.3357 | 22.119 | Arthropoda | - |
| scaffold435 | 2567289 | 0.3372 | 31.131 | Arthropoda | - |
| scaffold436 | 2543148 | 0.3451 | 23.139 | Arthropoda | - |
| scaffold437 | 2521720 | 0.3413 | 22.754 | Arthropoda | - |
| scaffold438 | 2573767 | 0.3434 | 21.665 | Arthropoda | - |
| scaffold439 | 2529208 | 0.3376 | 27.983 | Arthropoda | - |
| scaffold440 | 2518702 | 0.344 | 22.068 | Arthropoda | - |
| scaffold441 | 2436432 | 0.3415 | 21.734 | Arthropoda | - |
| scaffold442 | 2439127 | 0.3609 | 39.332 | Arthropoda | - |
| scaffold443 | 2411735 | 0.3417 | 28.21 | Arthropoda | - |
| scaffold444 | 2316748 | 0.3489 | 30.094 | Arthropoda | - |
| scaffold445 | 2321599 | 0.3429 | 31.508 | Arthropoda | - |
| scaffold446 | 2283111 | 0.3445 | 22.175 | Arthropoda | - |
| scaffold447 | 2257592 | 0.3352 | 21.851 | Arthropoda | - |
| scaffold448 | 2254340 | 0.342 | 22.288 | Arthropoda | - |
| scaffold449 | 2285011 | 0.3583 | 29.485 | Arthropoda | - |
| scaffold450 | 2223088 | 0.3617 | 27.199 | Arthropoda | - |
| scaffold451 | 2172026 | 0.3375 | 25.346 | Arthropoda | - |
| scaffold452 | 2140267 | 0.3567 | 27.225 | Arthropoda | - |
| scaffold453 | 2100522 | 0.3405 | 28.237 | Arthropoda | - |
| scaffold454 | 2112842 | 0.3442 | 24.196 | Arthropoda | - |
| scaffold455 | 2088110 | 0.3504 | 26.056 | Arthropoda | - |
| scaffold456 | 2077278 | 0.3472 | 31.034 | Arthropoda | - |
| scaffold457 | 2052519 | 0.345 | 27.517 | Arthropoda | - |
| scaffold458 | 2061175 | 0.3545 | 21.268 | Arthropoda | - |
| scaffold459 | 2045458 | 0.3379 | 23.996 | Arthropoda | - |
| scaffold460 | 2019400 | 0.3466 | 35.92 | Arthropoda | - |
| scaffold461 | 1976863 | 0.3418 | 33.886 | Arthropoda | - |
| scaffold462 | 1986977 | 0.3451 | 22.776 | Arthropoda | - |
| scaffold463 | 1948487 | 0.3397 | 25.969 | Arthropoda | - |
| scaffold464 | 1960870 | 0.3396 | 25.695 | Arthropoda | - |
| scaffold465 | 1940888 | 0.3353 | 34.671 | Arthropoda | - |
| scaffold466 | 1926498 | 0.3593 | 26.379 | Arthropoda | - |
| scaffold467 | 32379 | 0.3567 | 10.8 | no-hit | - |
| scaffold468 | 1874881 | 0.3463 | 21.952 | Arthropoda | - |
| scaffold469 | 1911057 | 0.3374 | 22.087 | Arthropoda | - |
| scaffold470 | 1842775 | 0.3651 | 27.397 | Arthropoda | - |
| scaffold471 | 1852878 | 0.35 | 29.689 | Arthropoda | - |
| scaffold472 | 1891582 | 0.3433 | 30.628 | Arthropoda | - |
| scaffold473 | 1874236 | 0.3414 | 23.539 | Arthropoda | - |
| scaffold474 | 1844553 | 0.3402 | 32.11 | Arthropoda | - |
| scaffold475 | 1846000 | 0.3582 | 21.941 | Arthropoda | - |
| scaffold476 | 1891836 | 0.335 | 36.396 | Arthropoda | - |
| scaffold477 | 1782482 | 0.3441 | 28.897 | Arthropoda | - |
| scaffold478 | 1831631 | 0.3464 | 22.992 | Arthropoda | - |
| scaffold479 | 1826129 | 0.3478 | 22.41 | Arthropoda | - |
| scaffold480 | 1800136 | 0.3408 | 35.201 | Arthropoda | - |
| scaffold481 | 1803250 | 0.3396 | 23.986 | Arthropoda | - |
| scaffold482 | 1783287 | 0.3429 | 25.524 | Arthropoda | - |
| scaffold483 | 1820712 | 0.3383 | 21.702 | Arthropoda | - |
| scaffold484 | 1758372 | 0.3382 | 25.372 | Arthropoda | - |
| scaffold485 | 1750706 | 0.3486 | 21.465 | Arthropoda | - |
| scaffold486 | 1758456 | 0.3527 | 37.873 | Arthropoda | - |
| scaffold487 | 1749500 | 0.3447 | 31.738 | Arthropoda | - |
| scaffold488 | 823220 | 0.3439 | 21.982 | Arthropoda | - |
| scaffold489 | 1687241 | 0.3426 | 28.411 | Arthropoda | - |
| scaffold490 | 1702223 | 0.3584 | 22.025 | Arthropoda | - |
| scaffold491 | 1670029 | 0.3431 | 59.386 | Arthropoda | - |
| scaffold492 | 1648146 | 0.3498 | 22.936 | Arthropoda | - |
| scaffold493 | 1673201 | 0.3683 | 25.005 | Arthropoda | - |
| scaffold494 | 1775488 | 0.3405 | 27.518 | Arthropoda | - |
| scaffold495 | 1639780 | 0.3506 | 25.02 | Arthropoda | - |
| scaffold496 | 1628501 | 0.3534 | 25.065 | Arthropoda | - |
| scaffold497 | 1620305 | 0.3454 | 22.935 | Arthropoda | - |
| scaffold498 | 1572706 | 0.3424 | 25.304 | Arthropoda | - |
| scaffold499 | 1585035 | 0.337 | 37.208 | Arthropoda | - |
| scaffold500 | 1608610 | 0.3422 | 25.327 | Arthropoda | - |
| scaffold501 | 1582630 | 0.3599 | 21.696 | Arthropoda | - |
| 000538F | 372108 | 0.3357 | 1786.322 | Proteobacteria | Discarded |
| 000574F | 321128 | 0.3364 | 2418.257 | Proteobacteria | Discarded |
| 000603F | 265373 | 0.3373 | 1151.135 | Proteobacteria | Discarded |
| 000610F | 239559 | 0.3386 | 1641.742 | Proteobacteria | Discarded |
| 000615F | 255025 | 0.3336 | 1401.391 | Proteobacteria | Discarded |
| 000618F | 246466 | 0.3377 | 1551.142 | Proteobacteria | Discarded |
| 000656F | 198562 | 0.3348 | 2327.206 | Proteobacteria | Discarded |
| 000660F | 197470 | 0.5883 | 9.304 | Proteobacteria | Discarded |
| 000666F | 181784 | 0.3427 | 2532.66 | Proteobacteria | Discarded |
| 000688F | 176603 | 0.3344 | 1768.192 | Proteobacteria | Discarded |
| 000707F | 165245 | 0.3318 | 1428.002 | Proteobacteria | Discarded |
| 000709F | 163052 | 0.3351 | 1227.067 | Proteobacteria | Discarded |
| 000826F | 122353 | 0.3498 | 2744.161 | Proteobacteria | Discarded |
| 000927F | 105235 | 0.5853 | 5.59 | no-hit | Discarded |
| 001127F | 77785 | 0.3836 | 106.09 | Firmicutes | Discarded |
| 001208F | 70203 | 0.3914 | 129.687 | Firmicutes | Discarded |
| 001293F | 44482 | 0.6516 | 58.273 | Proteobacteria | Discarded |
| 001341F | 60775 | 0.3742 | 120.219 | Firmicutes | Discarded |
| 001356F | 60114 | 0.5877 | 9.649 | Proteobacteria | Discarded |
| 001365F | 59677 | 0.3779 | 107.378 | Firmicutes | Discarded |
| 001374F | 59122 | 0.3689 | 114.455 | Firmicutes | Discarded |
| 001457F | 54234 | 0.3778 | 159.901 | Firmicutes | Discarded |
| 001473F | 53057 | 0.3961 | 133.916 | Firmicutes | Discarded |
| 001659F | 44189 | 0.3812 | 136.524 | Firmicutes | Discarded |
| 002069F | 30680 | 0.3243 | 15.343 | Bacteroidetes | Discarded |
| 002146F | 28446 | 0.42 | 292.433 | Firmicutes | Discarded |
| 002454F | 21043 | 0.3763 | 111.482 | Firmicutes | Discarded |
| 002707F | 15742 | 0.3918 | 104.729 | Firmicutes | Discarded |
| 002712F | 15561 | 0.3694 | 153.083 | Firmicutes | Discarded |
| 002751F | 14933 | 0.3502 | 114.589 | Firmicutes | Discarded |
| 002817F | 13753 | 0.3103 | 44.562 | Bacteroidetes | Discarded |
| 002825F | 13640 | 0.3261 | 3.607 | Viruses-undef | Discarded |
| 003156F | 7590 | 0.3308 | 127.891 | no-hit | Discarded |
| 003413F | 2417 | 0.6802 | 6.799 | Proteobacteria | Discarded |
| 3495 | 152396 | 0.5958 | 15.496 | Viruses-undef | Discarded |
| 3503 | 223561 | 0.5279 | 40.323 | Proteobacteria | Discarded |
| 001963F | 33520 | 0.2308 | 1788.685 | Mitochondria | Discarded |
| 002234F | 26250 | 0.2434 | 1697.818 | Mitochondria | Discarded |
| 002567F | 18505 | 0.226 | 1700.467 | Mitochondria | Discarded |
| 002824F | 13580 | 0.208 | 1674.749 | Mitochondria | Discarded |
| 002996F | 10264 | 0.2032 | 368.062 | Mitochondria | Discarded |
| 003115F | 8357 | 0.2003 | 600.416 | Mitochondria | Discarded |
| 003237F | 6103 | 0.2466 | 878.312 | Mitochondria | Discarded |
| 003247F | 5951 | 0.219 | 707.575 | Mitochondria | Discarded |
| 003258F | 5692 | 0.2841 | 1118.347 | Mitochondria | Discarded |
| 003271F | 5445 | 0.2279 | 638.694 | Mitochondria | Discarded |
| 003342F | 3837 | 0.2153 | 317.719 | Mitochondria | Discarded |
| 003343F | 3834 | 0.2149 | 415.522 | Mitochondria | Discarded |
| 003346F | 3819 | 0.216 | 277.752 | Mitochondria | Discarded |
| 003353F | 3737 | 0.2984 | 1110.22 | Mitochondria | Discarded |
| 003387F | 3019 | 0.2968 | 119.954 | Mitochondria | Discarded |
| 003397F | 2790 | 0.219 | 302.147 | Mitochondria | Discarded |
| 003449F | 1718 | 0.2421 | 17.343 | Mitochondria | Discarded |
| 003450F | 1702 | 0.228 | 8.321 | Mitochondria | Discarded |
| 003460F | 1547 | 0.1881 | 1183.075 | Mitochondria | Discarded |
| 003477F | 876 | 0.1712 | 1094.408 | Mitochondria | Discarded |
| 3507 | 17936 | 0.23 | 20.318 | Mitochondria | Discarded |
| 3519 | 18100 | 0.2307 | 1912.841 | Mitochondria | Discarded |
