# Supplementary figures and images for: Long-read sequence assembly of the firefly Pyrocoelia pectoralis genome
Source: Gigascience. 2017 Nov 24;6(12):1–7. doi: 10.1093/gigascience/gix112 (PMC5751067; doi:10.1093/gigascience/gix112)

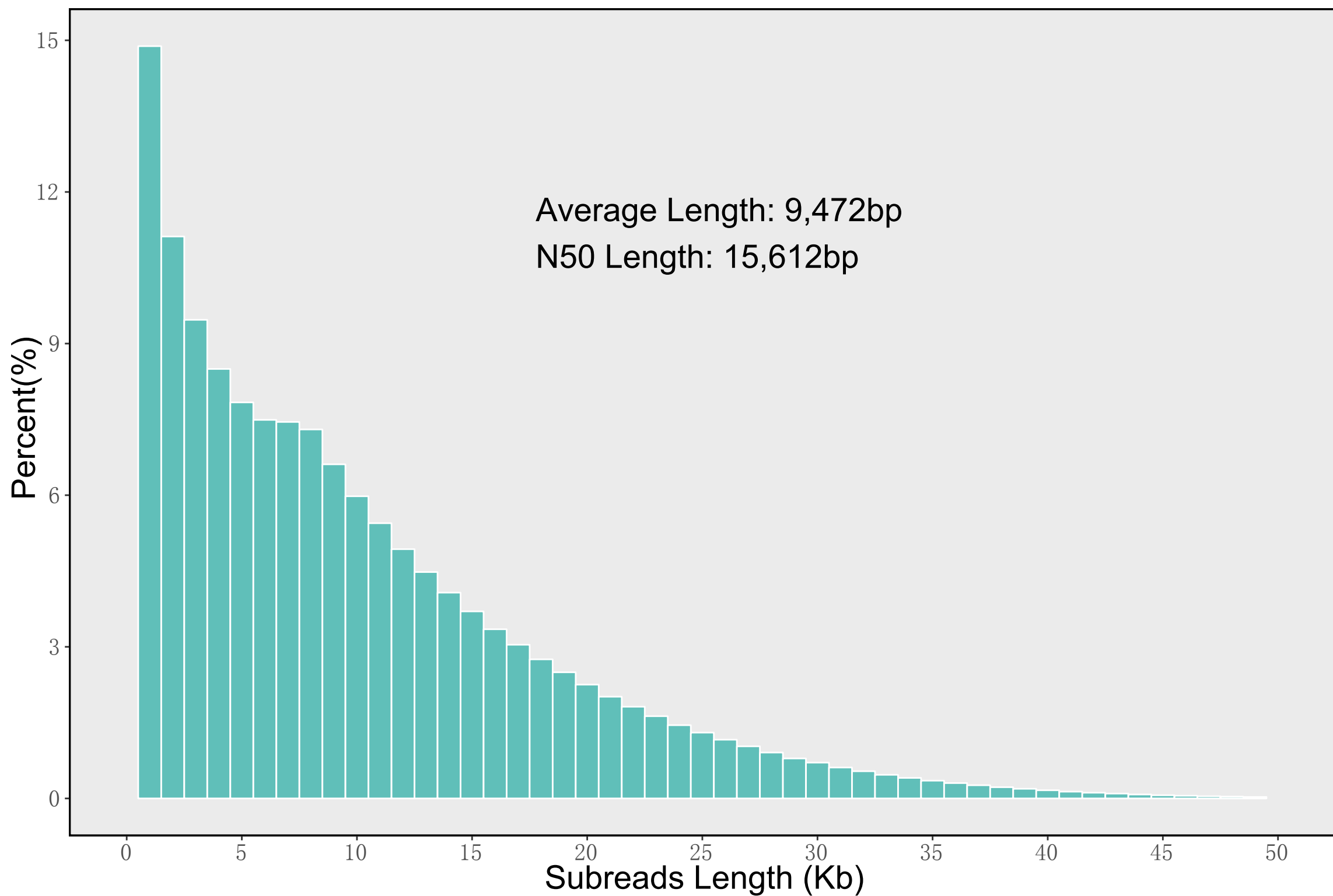

Supplement: Supplemental material [file gix112_supp.zip › Figure S1.pdf]

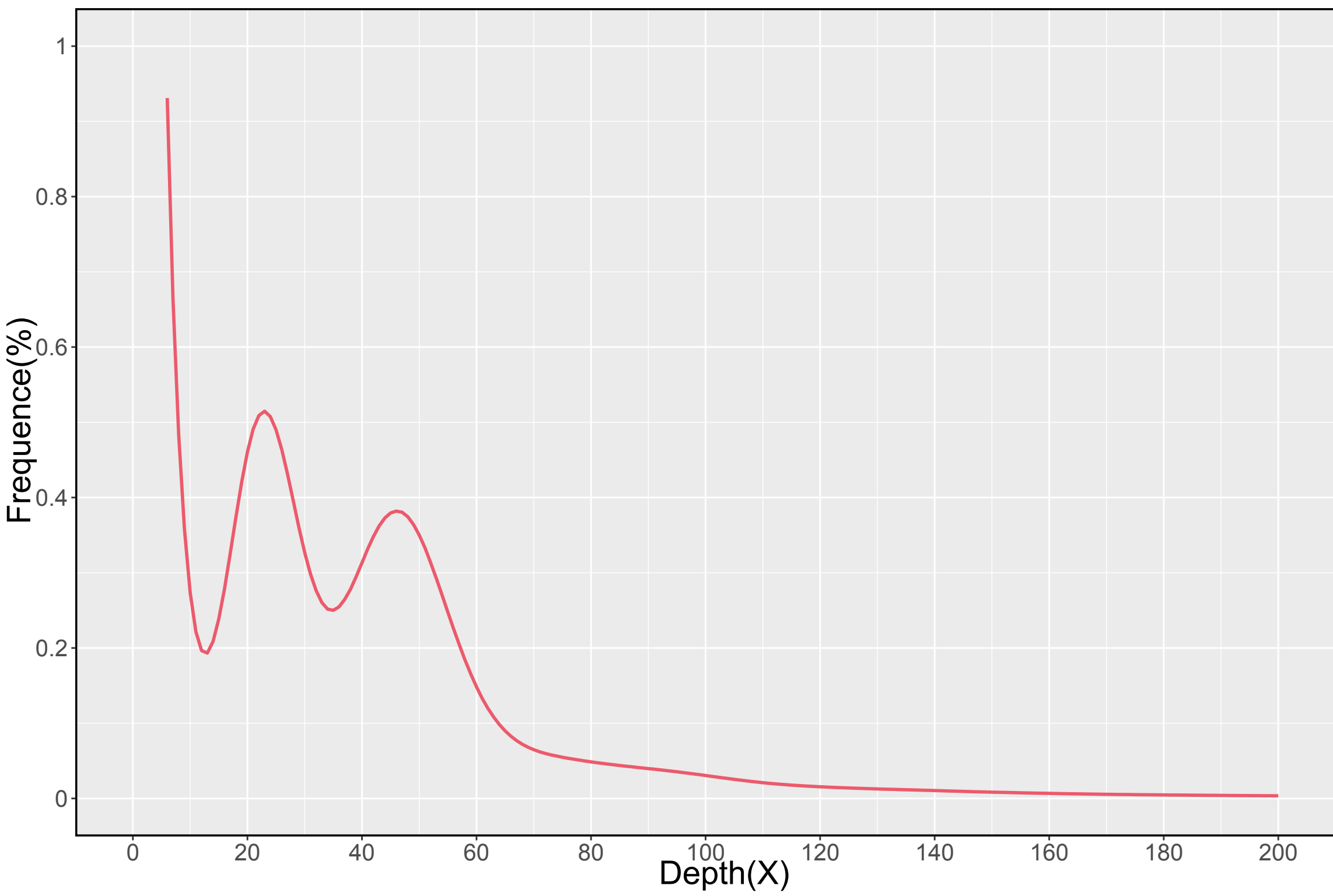

Supplement: Supplemental material [file gix112_supp.zip › Figure S2.pdf]

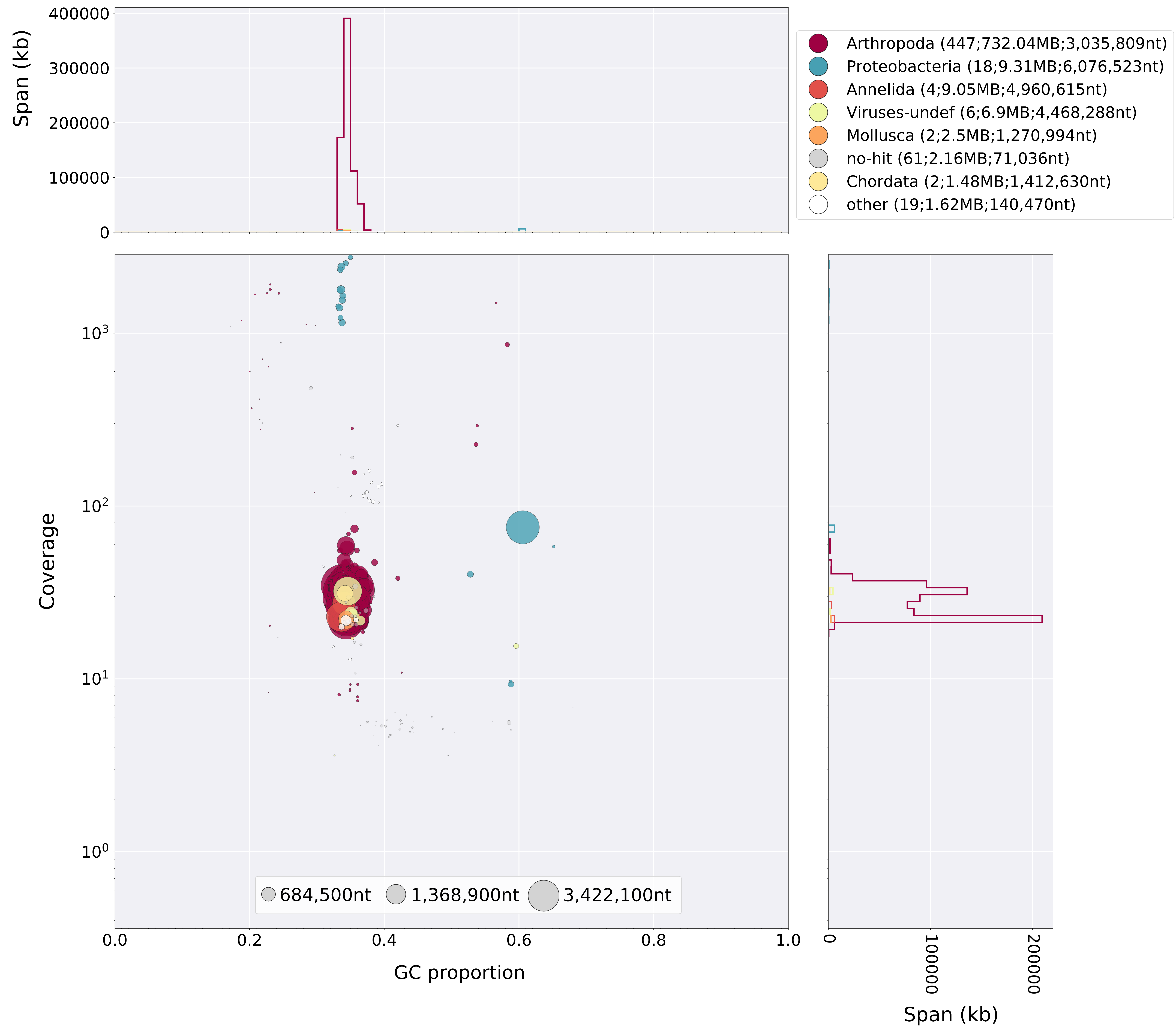

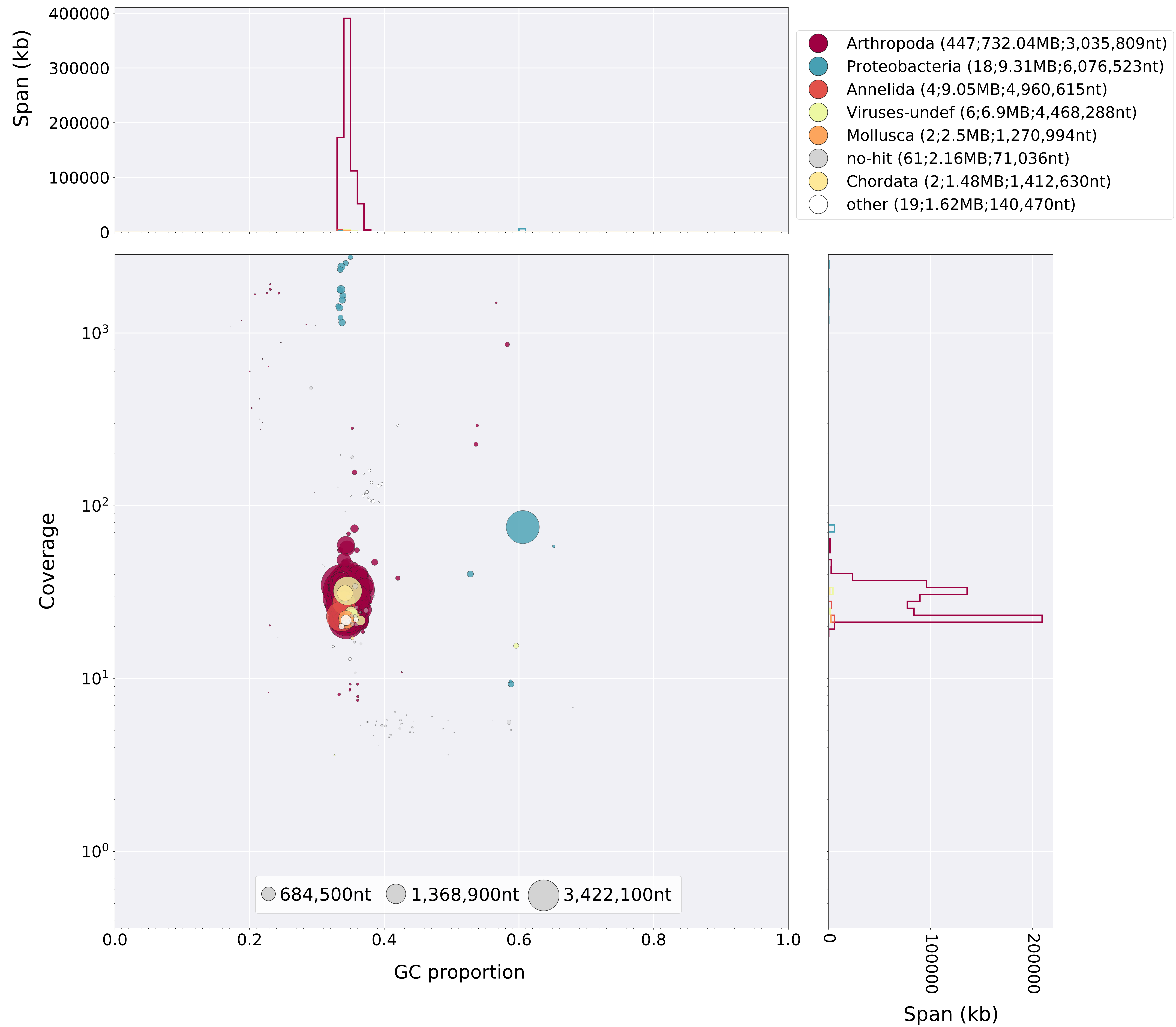

Supplement: Supplemental material [file gix112_supp.zip › Figure S3.pdf]
