# Supplementary material for: Long-read sequence assembly of the firefly Pyrocoelia pectoralis genome
Source: Gigascience. 2017 Nov 24;6(12):1–7. doi: 10.1093/gigascience/gix112 (PMC5751067; doi:10.1093/gigascience/gix112)
Supplement: Response-to-Reviewer-Comments_Original-Submission.pdf [file gix112_response-to-reviewer-comments_original-submission.pdf]

Author's Response To Reviewer Comments

Dear Editor and Reviewers:
Thank you for your letter and the reviewers' comments, we have studied comments carefully and made some updates and corrections in the paper. The followings are the responds to the reviewers' comments:

Reviewer reports:

Reviewer #2:
1. You mention that this is a gold-standard reference - but you don't define the term anywhere or cite a definition. (I know of old sanger-sequencing references for the terms complete, finished and draft genomes (PMID:12426325) and i agree that this is a very good assembly but am not sure I would say Gold-standard unless that has a technical definition)

Response: We have modified this term (a gold-standard reference) to a high-quality reference genome (see line 202, 283).

2. The only check for microbial contamination was a search against NCBI nt and mapping of RNA reads (Lines 169-175). I would argue that this is not sufficient for checking contamination, and it may lead you to throw away real instances of horizontal gene transfer. I strongly believe you should use read coverage of the contigs as well (eg BlobTools - http://dx.doi.org/10.12688/f1000research.12232.1 or Anvi'o https://doi.org/10.7717/peerj.1319 or CONCOCT doi.org/10.1038/nmeth.3103). I would have run blobtools for your data but I could not access the raw reads for SRP114311 or PRJNA394639

Response: We have analyzed potential contaminated contigs with BlobTools, and the identified potential bacterial or viral contigs were consistent with our previous result (see line 176-178, Fig. S3, Table S4).

- In the supp file letters seem to have jumped around (eg Line 13 "suggestinIllumina HiSeq X Teng"). Can you please check this is ok before final approval?

Response: Sorry about this errors and have checked.

53: change belongs to aquatic -> are aquatic

Response: This has been corrected as suggested (line 53).

60: harvest-trade - my apologies but I don't know what this term means and it is possible other readers won't either.

Response: This has been modified as "commercial harvesting and trade" (line 60).

66. Change sentence to: However, even with so

Response: This has been corrected as suggested (line 66).

73: Thanks very much for the protocols.io link. That's a great way to share the methods!

Response: Thanks.

85: Change within to with

Response: This has been corrected as suggested (line85).

88: Text says 9.5Kb but table says 11,285?

Response: 11 285 bp from Table S1 is basing on raw reads (polymerase reads) for PacBio Sequel system, while 9.5kb from manuscript is basing on subreads for PacBio Sequel system, we have added a comment at the bottom of the Table S1.
Polymerase read (formerly called "read"): A sequence of nucleotides incorporated by the DNA polymerase while reading a template, such as a circular SMRTbell™ template;
Subread: Each polymerase read is partitioned to form one or more subreads, which contain sequence from a single pass of a polymerase on a single strand of an insert within a SMRTbell™ template and no adapter sequences, the subreads were used to do assembly.

106: Number is unclear - with so many commas

Response: The commas have been replaced with blanks (line 106, 107)

134-138: I'm not sure I understand how Samtools and FreeBayes were used to do this.

Response: More details (bwa (v0.7.12) was used to map short reads to the error-corrected assembly and SAMtools (v0.1.19) and FreeBayes (v0.9.14) with default parameters under the diploid model were used to call snvs) about this method have been added to this part (line 134-139).

158-168: To collapse heterozygous regions, you cite Ref 18, but I could only find a conference poster abstract at http://eccb.iscb.org/2014/95/index.html - is there a better description/publication? The abstract says they use coverage but your scripts provided in the online folder don't seem to use read coverage as an input. I could have misread the scripts, so please correct me if I've gotten this wrong.

Response: Yes, we actually used a whole genome alignment (WGA) strategy to recognize and selectively remove alternative heterozygous contigs, ref 18 was used to state a standard assembly process tends to collapse homozygous regions and report heterozygous regions in alternative contigs. To avoid misunderstanding for this, we have updated a ref 19 (Pryszcz LP, Gabaldón T. Redundans: an assembly pipeline for highly heterozygous genomes. Nucleic Acids Res. 2016;44:e113–e113.) to explain this method we used (line 141).

191: Change to : 21 fold longer

Response: This has been corrected as suggested (line 194).

252: The files supplied suggest that the number of genes = number of transcripts = 23109. This seems unusual as MAKER is able to use transcripts to produce alternate transcripts for a gene. Could you share the MAKER command used (and include that in the supplemental info?)

Response: Because we set the parameter alt_splice=0 #Take extra steps to try and find alternative splicing, 1 = yes, 0 = no, which means maker did not produce alternative splicing for each gene. More parameters about maker have been added to the supplemental info (Additional File 1).

Fig 3 - I would add "proportion of BUSCO genes fully recovered" as the axis title (explained in the legend, but the axis label confused me at first glance)

Response: This has been corrected as suggested.

Reviewer #3: The high-quality draft genome produced will clearly be useful for exploring this family of insects. In their manuscript, Fu et al. present the genome of the firefly Pyrocoelia pectoralis. The sequencing data were obtained from the PacBio Sequel platform, and Illumina reads were generated for correcting the PacBio reads. In this Data Note, authors report statistics of the PacBio-generated assembly, assessment of genome completeness, and its annotation. The approaches used appear robust (except possibly for the removal of sequence contamination), the descriptions and interpretations are generally clear.

Regarding the removal of contaminant contigs, it would be good if authors provide a supplementary table with information on the removed scaffolds/contigs and the putative origin of these sequences, e.g. applying "blobtools" to generate Taxon-Annotated-GC-Coverage plots (TAGC plots) to visualise the contents of genome assembly as an essential QC step (see https://github.com/DRL/blobtools and Kumar, S., Jones, M., Koutsovoulos, G., Clarke, M. & Blaxter, M. Blobology: exploring raw genome data for contaminants, symbionts and parasites using taxon-annotated GC-coverage plots. Front Genet 4, 2013).

Response: We have analyzed potential contaminated contigs with BlobTools, and the identified potential bacterial or viral contigs were consistent with our previous result (see line 176-177, Fig. S3, Table S4).

Authors should assemble and provide the mitochondrial genome of P. pectoralis.

Response: This mitochondrial sequencings have been uploaded to the GigaScience database.

The authors used transcriptomic data for the assessment of genome completeness and for gene prediction but did not cite the corresponding study. Please cite:
Wang, K., Hong, W., Jiao, H. & Zhao, H. Transcriptome sequencing and phylogenetic analysis of four species of luminescent beetles. Scientific Reports 7, 1814 (2017)

Response: This has been corrected as suggested (line 258, ref 44).

The analysis of microsatellites sequences with the identification of 135,623 SSRs add little value (if none) to the study in the current state. Could these SSRs be useful for population genetic studies? If so, authors should provide at least a table with the most promising microsatellite loci, their coordinates on the genome and additional information that can be useful to infer their utility for population genetic studies (e.g. inter/intra-genic, part of transposable elements). A fasta file with the repetitive sequences and their flanking regions could be provide as well. This data set will be very useful to other researchers, as it will be ready to be used for designing primers for the screening and validation of these putative SSRs, before they are used as genetic markers in population genetic studies.

Response: Thank you for the suggestion, 2242 SSRs were selected as potential genetic markers in population genetic studies using strict standards, the details about coordinates, repeat unit, loci have been shown in Additional File 2. The repetitive sequences and their flanking regions have been uploaded to the GigaScience database (line 217-225).
