# Supplementary material for: Long-read sequence assembly of the firefly Pyrocoelia pectoralis genome
Source: Gigascience. 2017 Nov 24;6(12):1–7. doi: 10.1093/gigascience/gix112 (PMC5751067; doi:10.1093/gigascience/gix112)
Supplement: Response-to-Reviewer-Comments_Revision-1.pdf [file gix112_response-to-reviewer-comments_revision-1.pdf]

Author's Response To Reviewer Comments

Dear Editor and Reviewers:
Thank you for your letter and the reviewers' comments, the followings are the responds to the reviewers' comments:

Reviewer reports:
Line 106: 3 7238 236 952 is still an unusual number format. Whole numbers are typically separated in 3s, so if this is 37 billion, it should be 37,238,236,952, I think.
Response: This has been corrected as suggested (line 106).

2. The complete BlobTools plot sent by email is very informative and helpful. I'm a bit mystified as the plot looks quite different from Fig S3. The current complete plot looks like what you'd expect to see, but now I'm not sure how Fig S3 was generated. Based on the attached table and plot, it is possible that many (several kb long) contigs with coverage below 10 on the blobplot should be discarded even though they have no blast hits to bacteria / or have hits to arthropoda. I'd like to be sure that all contaminating contigs have been excluded (maybe even expand Table S4 to include all assembly contigs and add an extra column saying which contigs were discarded).

Response: Thank you for the suggestion, the first plot was just for all removed contigs, but we have updated a new plot for all data. We have removed contigs with coverage below 10 on the blobplot or had the best hit to non-Arthropoda and without any transcript reads (not a single read, but a isoform can assembly from this contig with histat2) and homolog genes from BUSCO, because those contigs with transcript reads or homolog genes from BUSCO mapped might were real instances of horizontal gene transfer. Besides, we assembled the genome with PacBio long reads, which did not have the GC bias or the depth bias, but the illuminal reads had the GC bias or the depth bias, so those contigs with low depth might be caused by the bias, the Table S4 had showed all contigs and with an extra column saying whether discarded. We also updated all data in this Manuscript as the final genome had changed (line 170-181, Fig. S3, Table S4).
