# Supplementary material for: Long-read sequence assembly of the firefly Pyrocoelia pectoralis genome
Source: Gigascience. 2017 Nov 24;6(12):1–7. doi: 10.1093/gigascience/gix112 (PMC5751067; doi:10.1093/gigascience/gix112)
Supplement: Reviewer-1-Report-(Original-Submission).pdf [file gix112_reviewer-1-report-(original-submission).pdf]

**Reviewer Report**

**Title:** Long-read sequence assembly of the firefly Pyrocoelia pectoralis genome

**Version:** Original Submission **Date:** 14 Sep 2017

**Reviewer name:** Stephen Richards

**Reviewer Comments to Author:**

Good Job! - The authors are to be commended on doing an excellent job on this assembly, which will be part of the foundation for beetle, insect, arthropod and animal genomics as we move into the future. We have all been moving into a world of higher quality assemblies, and this work clearly increases the bar for the rest of us. There is a minor issue that perhaps more species protein sequences could be used for the Maker annotation,
but I do not think that should prevent publication. In the future I would recommend using many more species but in a non-redundant way to reduce compute costs.

So I put major revision for one reason and one reason only - I can't find the data in NCBI.
I did find the supporting reads in the reviewer link so that is great!
I could not find the BioProject - I guess it is hidden unit publication - but I can't review things I can't see.
In the manuscript it says that the assembly and gene models are in the giga science database - I hadn't used this one before - but it looks great. Unfortunately searching for Pyrocoelia didn't return anything, and I could not find an accession number in the manuscript.
My general aim as a reviewer for these papers is to check that I can download the sequence from NCBI, to check that the stats match (i.e. that the whole file is there) and to check that I can see the automated annotations.
I don't think that the Giga Science database alone is good enough - I think it also has to be in the 3 main genome databases - NCBI/EMBL-EBI/DDBJ (INSDC)- you only need to submit to one as they talk. - You did great work, show it off a little. The manuscript should have the accession number for the assembly in it.
So I am asking the editor to accept for publication as is, as soon as he or she confirms the assembly and ideally gene models have been put in the INSDC databases. There is no point coming back to me - if the editor sees the data in the databases, then immediately accept.

Otherwise it looks like great work!

Stephen Richards

ps - if you need a place for manual annotation of favorite genes using the webApollo tools - then perhaps the i5k workspace might be a nice place to host this dataset, if only for the nice browser.
This might be especially good if you have difficulties getting the annotation into NCBI etc in addition to

GigaDB.
If you are worried about getting scooped during the public submission process - perhaps bioarchive for the preprint? - but at this point I think you have nothing to worry about.

**Level of Interest**

Please indicate how interesting you found the manuscript: An article of importance in its field

**Quality of Written English**

Please indicate the quality of language in the manuscript: Acceptable

**Declaration of Competing Interests**

Please complete a declaration of competing interests, considering the following questions:

- Have you in the past five years received reimbursements, fees, funding, or salary from an organisation that may in any way gain or lose financially from the publication of this manuscript, either now or in the future?
- Do you hold any stocks or shares in an organisation that may in any way gain or lose financially from the publication of this manuscript, either now or in the future?
- Do you hold or are you currently applying for any patents relating to the content of the manuscript?
- Have you received reimbursements, fees, funding, or salary from an organization that holds or has applied for patents relating to the content of the manuscript?
- Do you have any other financial competing interests?
- Do you have any non-financial competing interests in relation to this paper?

If you can answer no to all of the above, write 'I declare that I have no competing interests' below. If your reply is yes to any, please give details below.

I declare that I have no competing interests

I agree to the open peer review policy of the journal. I understand that my name will be included on my report to the authors and, if the manuscript is accepted for publication, my named report including any attachments I upload will be posted on the website along with the authors' responses. I agree for my report to be made available under an Open Access Creative Commons CC-BY license (http://creativecommons.org/licenses/by/4.0/). I understand that any comments which I do not wish to

be included in my named report can be included as confidential comments to the editors, which will not be published.

I agree to the open peer review policy of the journal
