# Supplementary material for: Long-read sequence assembly of the firefly Pyrocoelia pectoralis genome
Source: Gigascience. 2017 Nov 24;6(12):1–7. doi: 10.1093/gigascience/gix112 (PMC5751067; doi:10.1093/gigascience/gix112)
Supplement: Reviewer-2-Report-(Original-Submission).pdf [file gix112_reviewer-2-report-(original-submission).pdf]

Reviewer Report

Title: Long-read sequence assembly of the firefly Pyrocoelia pectoralis genome

Version: Original Submission **Date:** 17 Sep 2017

Reviewer name: Sujai Kumar

Reviewer Comments to Author:

Dear Authors

My sincere apologies for taking so long to return this review.

First of all - congratulations on a great assembly that shows the power of long read sequencing. I think this is a great example of a genome announcement and can be a good model for other long read assembly papers.

However, I have a few general and specific comments that would need to be addressed before publication:

General comments:

1. You mention that this is a gold-standard reference - but you don't define the term anywhere or cite a definition. (I know of old sanger-sequencing references for the terms complete, finished and draft genomes (PMID:12426325) and i agree that this is a very good assembly but am not sure I would say Gold-standard unless that has a technical definition)

2. The only check for microbial contamination was a search against NCBI nt and mapping of RNA reads (Lines 169-175). I would argue that this is not sufficient for checking contamination, and it may lead you to throw away real instances of horizontal gene transfer. I strongly believe you should use read coverage of the contigs as well (eg BlobTools - http://dx.doi.org/10.12688/f1000research.12232.1 or Anvi'o https://doi.org/10.7717/peerj.1319 or CONCOCT doi.org/10.1038/nmeth.3103). I would have run blobtools for your data but I could not access the raw reads for SRP114311 or PRJNA394639

Specific comments:

- In the supp file letters seem to have jumped around (eg Line 13 "suggestinIllumina HiSeq X Teng"). Can you please check this is ok before final approval?
Line numbers:

53: change belongs to aquatic -> are aquatic

60: harvest-trade - my apologies but I don't know what this term means and it is possible other readers won't either.

66. Change sentence to: However, even with so

73: Thanks very much for the protocols.io link. That's a great way to share the methods!

85: Change within to with

88: Text says 9.5Kb but table says 11,285?

106: Number is unclear - with so many commas

134-138: I'm not sure I understand how Samtools and FreeBayes were used to do this.

158-168: To collapse heterozygous regions, you cite Ref 18, but I could only find a conference poster abstract at http://eccb.iscb.org/2014/95/index.html - is there a better description/publication? The abstract says they use coverage but your scripts provided in the online folder don't seem to use read coverage as an input. I could have misread the scripts, so please correct me if I've gotten this wrong.

191: Change to : 21 fold longer

252: The files supplied suggest that the number of genes = number of transcripts = 23109. This seems unusual as MAKER is able to use transcripts to produce alternate transcripts for a gene. Could you share the MAKER command used (and include that in the supplemental info?)

Fig 3 - I would add "proportion of BUSCO genes fully recovered" as the axis title (explained in the legend, but the axis label confused me at first glance)

Best wishes,

Sujai

Level of Interest

Please indicate how interesting you found the manuscript: An article whose findings are important to those with closely related research interests

Quality of Written English

Please indicate the quality of language in the manuscript: Not suitable for publication unless extensively edited

Declaration of Competing Interests

Please complete a declaration of competing interests, considering the following questions:

- Have you in the past five years received reimbursements, fees, funding, or salary from an organisation that may in any way gain or lose financially from the publication of this manuscript, either now or in the future?
- Do you hold any stocks or shares in an organisation that may in any way gain or lose financially from the publication of this manuscript, either now or in the future?
- Do you hold or are you currently applying for any patents relating to the content of the manuscript?
- Have you received reimbursements, fees, funding, or salary from an organization that holds or has applied for patents relating to the content of the manuscript?
- Do you have any other financial competing interests?
- Do you have any non-financial competing interests in relation to this paper?

If you can answer no to all of the above, write 'I declare that I have no competing interests' below. If your reply is yes to any, please give details below.

I declare that I have no competing interests

I agree to the open peer review policy of the journal. I understand that my name will be included on my report to the authors and, if the manuscript is accepted for publication, my named report including any attachments I upload will be posted on the website along with the authors' responses. I agree for my report to be made available under an Open Access Creative Commons CC-BY license (http://creativecommons.org/licenses/by/4.0/). I understand that any comments which I do not wish to be included in my named report can be included as confidential comments to the editors, which will not be published.

I agree to the open peer review policy of the journal
