# Supplementary material for: Long-read sequence assembly of the firefly Pyrocoelia pectoralis genome
Source: Gigascience. 2017 Nov 24;6(12):1–7. doi: 10.1093/gigascience/gix112 (PMC5751067; doi:10.1093/gigascience/gix112)
Supplement: Reviewer-2-Report-(Revision-1).pdf [file gix112_reviewer-2-report-(revision-1).pdf]

Reviewer Report

Title: Long-read sequence assembly of the firefly Pyrocoelia pectoralis genome

Version: Revision 1 **Date:** 16 Oct 2017

Reviewer name: Sujai Kumar

Reviewer Comments to Author:

Dear Authors

Many thanks for your updated manuscript. I agree with all of your revisions, with the exception of two things:

1. Line 106: 3 7238 236 952 is still an unusual number format. Whole numbers are typically separated in 3s, so if this is 37 billion, it should be 37,238,236,952, I think.

2. The complete BlobTools plot sent by email is very informative and helpful. I'm a bit mystified as the plot looks quite different from Fig S3. The current complete plot looks like what you'd expect to see, but now I'm not sure how Fig S3 was generated.

Based on the attached table and plot, it is possible that many (several kb long) contigs with coverage below 10 on the blobplot should be discarded even though they have no blast hits to bacteria / or have hits to arthropoda. I'd like to be sure that all contaminating contigs have been excluded (maybe even expand Table S4 to include all assembly contigs and add an extra column saying which contigs were discarded).

Sorry to be so picky, but after tardigate I'm even more aware of how mislabelled contigs deposited in the public databases can cause problems (not so much for the findings of the current study, but for future studies that may have hits to the mislabelled contigs)

These are minor things however, and will only change the final fasta file, and maybe a few numbers in tables. Otherwise the manuscript is ready to go.

Best wishes,

- Sujai

Level of Interest

Please indicate how interesting you found the manuscript: An article whose findings are important to those with closely related research interests

Quality of Written English

Please indicate the quality of language in the manuscript: Acceptable

Declaration of Competing Interests

Please complete a declaration of competing interests, considering the following questions:

- Have you in the past five years received reimbursements, fees, funding, or salary from an organisation that may in any way gain or lose financially from the publication of this manuscript, either now or in the future?
- Do you hold any stocks or shares in an organisation that may in any way gain or lose financially from the publication of this manuscript, either now or in the future?
- Do you hold or are you currently applying for any patents relating to the content of the manuscript?
- Have you received reimbursements, fees, funding, or salary from an organization that holds or has applied for patents relating to the content of the manuscript?
- Do you have any other financial competing interests?
- Do you have any non-financial competing interests in relation to this paper?

If you can answer no to all of the above, write 'I declare that I have no competing interests' below. If your reply is yes to any, please give details below.

I declare that I have no competing interests

I agree to the open peer review policy of the journal. I understand that my name will be included on my report to the authors and, if the manuscript is accepted for publication, my named report including any attachments I upload will be posted on the website along with the authors' responses. I agree for my report to be made available under an Open Access Creative Commons CC-BY license (http://creativecommons.org/licenses/by/4.0/). I understand that any comments which I do not wish to be included in my named report can be included as confidential comments to the editors, which will not be published.

I agree to the open peer review policy of the journal
