# Supplementary material for: Long-read sequence assembly of the firefly Pyrocoelia pectoralis genome
Source: Gigascience. 2017 Nov 24;6(12):1–7. doi: 10.1093/gigascience/gix112 (PMC5751067; doi:10.1093/gigascience/gix112)
Supplement: Reviewer-3-Report-(Original-Submission).pdf [file gix112_reviewer-3-report-(original-submission).pdf]

21 Sep 2017

Reviewer Report

Title: Long-read sequence assembly of the firefly Pyrocoelia pectoralis genome

Version: Original Submission **Date:** 21 Sep 2017

Reviewer name: Evgeny Zdobnov

Reviewer Comments to Author:

The high-quality draft genome produced will clearly be useful for exploring this family of insects. In their manuscript, Fu et al. present the genome of the firefly Pyrocoelia pectoralis. The sequencing data were obtained from the PacBio Sequel platform, and Illumina reads were generated for correcting the PacBio reads. In this Data Note, authors report statistics of the PacBio-generated assembly, assessment of genome completeness, and its annotation. The approaches used appear robust (except possibly for the removal of sequence contamination), the descriptions and interpretations are generally clear.

Regarding the removal of contaminant contigs, it would be good if authors provide a supplementary table with information on the removed scaffolds/contigs and the putative origin of these sequences, e.g. applying "blobtools" to generate Taxon-Annotated-GC-Coverage plots (TAGC plots) to visualise the contents of genome assembly as an essential QC step (see https://github.com/DRL/blobtools and Kumar, S., Jones, M., Koutsovoulos, G., Clarke, M. & Blaxter, M. Blobology: exploring raw genome data for contaminants, symbionts and parasites using taxon-annotated GC-coverage plots. Front Genet 4, 2013).

Authors should assemble and provide the mitochondrial genome of P. pectoralis.

The authors used transcriptomic data for the assessment of genome completeness and for gene prediction but did not cite the corresponding study. Please cite:
Wang, K., Hong, W., Jiao, H. & Zhao, H. Transcriptome sequencing and phylogenetic analysis of four species of luminescent beetles. Scientific Reports 7, 1814 (2017)

The analysis of microsatellites sequences with the identification of 135,623 SSRs add little value (if none) to the study in the current state. Could these SSRs be useful for population genetic studies? If so, authors should provide at least a table with the most promising microsatellite loci, their coordinates on the genome and additional information that can be useful to infer their utility for population genetic studies (e.g. inter/intra-genic, part of transposable elements). A fasta file with the repetitive sequences and their flanking regions could be provide as well. This data set will be very useful to other researchers, as it will be ready to be used for designing primers for the screening and validation of these putative SSRs, before they are used as genetic markers in population genetic studies.

Level of Interest

Please indicate how interesting you found the manuscript: An article of importance in its field

Quality of Written English

Please indicate the quality of language in the manuscript: Acceptable

Declaration of Competing Interests

Please complete a declaration of competing interests, considering the following questions:

- Have you in the past five years received reimbursements, fees, funding, or salary from an organisation that may in any way gain or lose financially from the publication of this manuscript, either now or in the future?
- Do you hold any stocks or shares in an organisation that may in any way gain or lose financially from the publication of this manuscript, either now or in the future?
- Do you hold or are you currently applying for any patents relating to the content of the manuscript?
- Have you received reimbursements, fees, funding, or salary from an organization that holds or has applied for patents relating to the content of the manuscript?
- Do you have any other financial competing interests?
- Do you have any non-financial competing interests in relation to this paper?

If you can answer no to all of the above, write 'I declare that I have no competing interests' below. If your reply is yes to any, please give details below.

non

I agree to the open peer review policy of the journal. I understand that my name will be included on my report to the authors and, if the manuscript is accepted for publication, my named report including any attachments I upload will be posted on the website along with the authors' responses. I agree for my report to be made available under an Open Access Creative Commons CC-BY license (http://creativecommons.org/licenses/by/4.0/). I understand that any comments which I do not wish to be included in my named report can be included as confidential comments to the editors, which will not be published.

I agree to the open peer review policy of the journal
